# Extraction of 5-Hydroxymethylfurfural and Furfural in Aqueous Biphasic Systems: A COSMO-RS Guided Approach to Greener Solvent Selection

**DOI:** 10.1021/acssuschemeng.3c07894

**Published:** 2024-02-20

**Authors:** Dominik Soukup-Carne, Pablo López-Porfiri, Felipe Sanchez Bragagnolo, Cristiano Soleo Funari, Xiaolei Fan, María González-Miquel, Jesús Esteban

**Affiliations:** † Department of Chemical Engineering, The University of Manchester, Oxford Road, Manchester M13 9PL, United Kingdom; ‡ Multidisciplinary Laboratory of Food and Health (LabMAS), School of Applied Sciences (FCA), University of Campinas (UNICAMP), Rua Pedro Zaccaria 1300, 13484-350 Limeira, SP, Brazil; § Green Biotech Network, School of Agricultural Sciences, São Paulo State University, Av. Universitária 3780, Botucatu, 18610-034 São Paulo, Brazil; ∥ Department of Chemical and Environmental Engineering, ETSI Industriales, Universidad Politécnica de Madrid, José Gutiérrez Abascal 2, 28006 Madrid, Spain

**Keywords:** Furans, COSMO-RS, extraction, partition coefficient, green solvent, solvent recovery

## Abstract

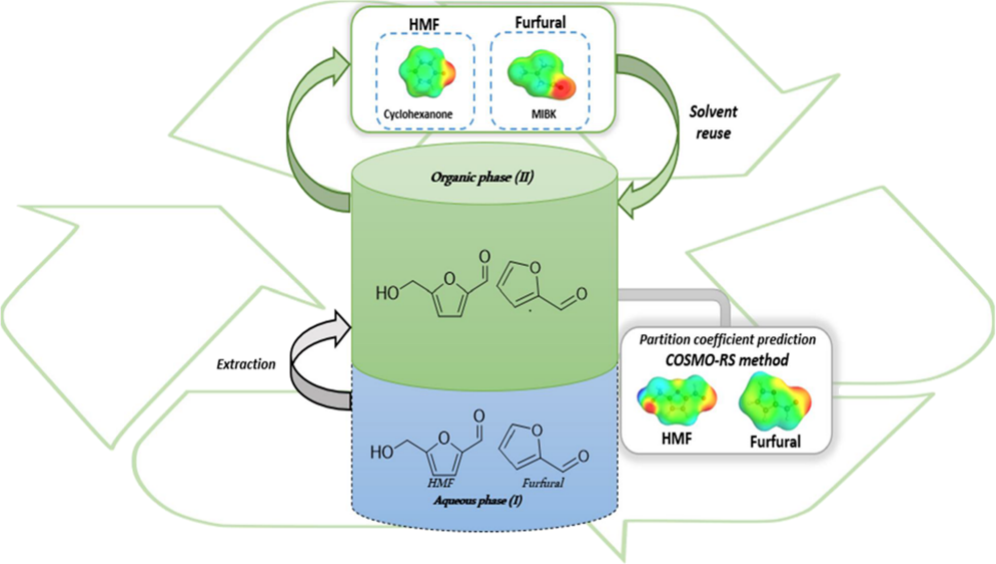

5-Hydroxymethylfurfural (HMF) and furfural (Fur) are promising biobased platform chemicals, derived from the dehydration of carbohydrate feedstocks, normally conducted in an aqueous phase. Plagued by side-reactions in such phase, such as the rehydration to levulinic acid (LA) and formic acid (FA) or self-condensation to humins, HMF and Fur necessitates diversification from monophasic aqueous reaction systems toward biphasic systems to mitigate undesired side-reactions. Here, a methodology based on the COnductor-like Screening MOdel for Real Solvents (COSMO-RS) method was used to screen solvent candidates based on the predicted partition coefficients (*K*_*i*_). Hansen solubility parameters in conjunction with excess thermodynamic quantities determined by COSMO-RS were employed to assess solvent compatibility. Experimental validation of the COSMO-RS values highlighted only minor deviations from the predictions with root-mean-square-error (RMSE) values of HMF and Fur at 0.76 and 5.32, respectively, at 298 K. The combined effort suggested cyclohexanone, isophorone, and methyl isobutyl ketone (MIBK) as the best candidates. Finally, extraction solvent reuse demonstrated cyclohexanone suitability for HMF extraction with *K*_*HMF*_ of 3.66 and MIBK for Fur with *K*_*Fur*_ 7.80 with consistent partitioning across four total runs. Both solvents are classified as recommended by the CHEM21 solvent selection guide, hence adding to the sustainability of the process.

## Abstract 2

Cleaner solvent selection and recycling for 5-hydroxymethylfurfural and furfural extraction are guided by computational approaches and experimentally validated.

## Introduction

**Figure 1:**
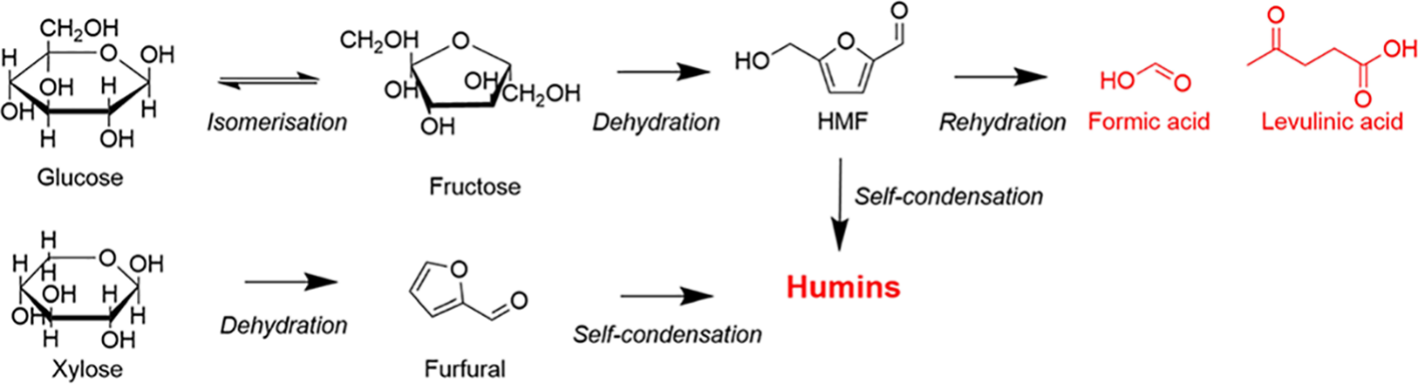
Reaction scheme of production of HMF and Fur from hexose and pentose sugars, respectively, under the influence of an acidic media, with products highlighted in red denoting undesired byproducts.

The ever-growing demand for fuels and chemicals necessitates the diversification of feedstocks to mitigate the long-term effects of the overconsumption of fossil-based resources. Biorefinery concepts around the use of lignocellulosic biomass present an alternative for integrated sustainable chemical production. The US Department of Energy (DoE) aired a list of top biobased platform chemical candidates,^1^ including 5-hydroxymethylfurfural (HMF) and furfural (Fur). HMF is described as the “sleeping giant” of biobased platform chemicals, due to the vast array of potential derivatives possible through synthetic upgrading of the furan ring as well as the carbonyl and hydroxyl moieties.^2,3^ Possible derivatives include 2,5-dimethylfuran, furan dicarboxylic acid (FDCA), and 5-methylfurfural, which can be used as biofuels, polymer precursors, and synthetic intermediates, respectively.^4−6^ The majority of demand for Fur (c. 90%) is directly used for the production of furfuryl alcohol, which is predominantly used as a solvent.^7^ Typically, both HMF and Fur are produced in the presence of an acid catalyst in monophasic systems, generally aqueous reaction media through the dehydration of hexose and pentose sugars, respectively.^8,9^ Yields from monophasic systems of both HMF and Fur are relatively low due to potential side reactions that occur, such as rehydration of HMF toward levulinic acid (LA) and formic acid (FA) or the self-condensation of both HMF and Fur to insoluble humins, as shown in Figure 1.

Mitigation of the generation of these byproducts can be realized through the use of aqueous biphasic systems, wherein a nonpolar phase is utilized to perform the extraction as reaction progresses, which is seen as an approach to realizing process intensification.^10^ Henceforth, considerations on the performance and applicability of selected solvents must be understood, such as the separation performance, recyclability, and their environmental, health and safety (EHS) profile to develop techno-economically viable and sustainable processes.

Solvent selection to constitute the nonpolar phase is a matter of utmost relevance in this type of operation. Lately, *in silico* tools have provided a magnificent resource for solvent screening that allows fast identification of promising solvents for separations. One of such tools is the COnductor-like Screening MOdel for Real Solvents (COSMO-RS) method, which is an ab initio semiquantitative quasi quantum chemistry-based computational approach.^11−13^ This method has already seen success when applied to the case of solvent selection for the separation of HMF and Fur, with a survey of existing studies shown in Table S1. These works presented encompass both computational only studies and those with experimental validation of the COSMO-RS predicted partition coefficient for molecular solvents and hydrophobic deep eutectic solvents. Furthermore, both large scale and targeted screenings are also included. Two large scale screenings were performed by Blumenthal et al. and Wang et al., with 6000 and 2500 initial solvents screened for the extraction of HMF.^14,15^ These works identified top performers, *o*-propylphenol, *o*-isopropylphenol, and 3-chlorophenol, although it must be noted these are hazardous solvents under CHEM21 classification.^16^ Two authors additionally detailed the screening of solvents for HMF or Fur extraction, with 3-chlorophenol identified for both HMF and Fur extraction, ethyl acetate for HMF, and methyl propionate for Fur.^17,18^ Finally, for the studies focusing primarily on Fur extraction, thymol was identified from a selection of biobased and terpene-derived biocarbonate solvents.^19,20^ It is worthwhile remarking that the use of COSMO-RS has been expanded recently to include more novel solvents, namely, those using hydrophobic eutectic solvents as the extraction phase, which have been tested for HMF and Fur extraction.^21−23^ Nevertheless, these solvents are challenging to recycle by distillation owing to their low vapor pressure, which makes research on the use of molecular solvents still relevant if distillation is to be used as the recovery alternative. In addition, inclusion of electrolytes has also been observed to facilitate the partition of HMF in aqueous biphasic systems through the so-called salting-out effect, with this effect not being observed with Fur owing to its lack of a hydroxyl function. However, these electrolytes additives are known to cause deactivation in heterogeneous catalysts, hence not attracting as much interest as lean aqueous biphasic systems.^18,24−26^ Furthermore, the COSMO-RS method proves limited in modeling these systems, with other thermodynamic models with correction factors preferable such as ePC-SAFT and NRTL.^18,24−26^

Further computational predictive methods can be applied to solvent screening in the form of Hansen solubility parameters (HSP), which consist of three terms, dispersion (*δ*_*D*_), dipole moment (*δ*_*P*_), and hydrogen bond (*δ*_*H*_) to account for the corresponding solvent–solute interactions.^27,28^ Based on the concept of ‘like dissolves like’ and the similarity between the HSP of solute and solvents, it is possible to propose the similarity/solubility of specific targets in different molecular solvents or mixtures.^29^ Additionally, HSP have been utilized to relate the polarity of the reaction medium and the yield of Fur from xylose in mono- and biphasic systems.^30,31^ However, HSP can also be used to predict the likelihood of solute dissolution in a given solvent and can be used as an additional tool for the purpose of selecting an extracting agent.^29^

Not only do solvents have to perform the desired separation efficiently but also with an eye on green chemistry principles,^32^ the use of hazardous organic solvents should be avoided. For example, previous efforts on the biphasic production of HMF and Fur have used solvents such as toluene and dichloromethane^33,34^ or, in the case of the studies alluded to above with COSMO-RS screenings, phenol and halogenated derivatives.^14,15,17^ Solvent selection guides are useful tools in identifying less hazardous alternatives to commonly used extraction solvents.^16,35−39^ Specifically, the CHEM21 guide allows the estimation of EHS profiles.^18,40−42^ With ever growing pressure from legislation such as such as Registration, Evaluation, Authorization and Restriction of Chemicals (REACH EC 1907/2006) and Integrated Pollution Prevention and Control (IPPC, EC 1/2008), the identification of cleaner extraction media is all the more important.^43,44^

This work details a combined computational and experimental approach to propose greener solvent selection for the extraction of HMF and Fur from aqueous media for application in the biphasic production of Furans from sugars. This approach centered around the initial use of the COSMO-RS method to screen a selected pool of solvents, with consideration on EHS profiles based on the CHEM21 solvent selection guide directing the final set of solvents to be experimentally validated. This combined approach builds a general framework for the targeted selection of industrially relevant cleaner extraction solvents in aqueous biphasic nonsalted systems. As a result, experimental partition coefficients identified a selection of exceptional solvents to assess solvent reuse across multiple runs. In addition, COSMO-RS was utilized to calculate excess thermodynamic contributions and HSP was evaluated to provide insights into the solvent–solute interactions driving the partitioning observed. These findings can be used to guide process development for the production of Furans from biomass.

## Methods

### Materials

The solutes to be used in this study were HMF (CAS 67-47-0, ≥99.5%) and LA (CAS 123-76-2, ≥98%), both purchased from Fluorochem, Fur (CAS 98-01-1, ≥99%) from Sigma-Aldrich, and FA (CAS 64-18-6, ≥ 98%) from Fischer Scientific. The following compounds were utilized as solvents: MIBK (CAS 108-10-1, ≥99%) was purchased from Alfa Aesar, isophorone (CAS 78-59-1, ≥97%) and 4-isopropylphenol (CAS 99-89-8, ≥98%) from Thermo Scientific, cyclohexanone from Scientific Laboratory Supplies (CAS 108-94-1, ≥ 99%), methyl tetrahydrofuran (MTHF) (CAS 96-47-9, ≥99%, and triethylamine (CAS 121-44-8, ≥99%) from Apollo Scientific, dimethyl carbonate (DMC) (CAS 616-38-6, ≥99%) and cyclopentyl methyl ether (CPME) (CAS 5614-37-9, ≥99%) from Fluorochem, isopropyl alcohol (IPA) (67-63-0, ≥99.5%,) from Sigma-Aldrich, 1-octanol (CAS 111-87-5, ≥99%) from TCI and 1,2-dichloroethane (DCE) (CAS 107-06-2, ≥99.8%), and 1,2-ethanediol diacetate (CAS 11-55-7, ≥99%) from Thermo Scientific Acros. Finally, Milli-Q water was supplied by an Elga PureLab option Q DV25 at 18.2 mΩ.

### COSMO-RS Procedure for Solvent Selection

This work focuses on the screening of a select solvent pool to evaluate the process suitability of said solvents when applied to HMF and Fur extraction; hence, an initial set must be selected. This solvent set was selected through the combined use of solvent selection guides such as AstraZeneca, ACS, Sanofi, CHEM21, GSK, and Pfizer, in addition to the work of Moity et al.^16,35−39,45^ These combined sources yielded an initial set of 176 solvents to be screened using the COSMO-RS method comprising highly common solvents in industrial practice as well as biobased alternatives with an increasing interest in use and applicability on COSMO-RS. This included a range of varying functional groups such as alcohols (34), esters (48), ketones (9), organic acids (9), ethers (22), organic carbonates (4), dipolar aprotic (8), aromatics (6), hydrocarbons (12), halogenated (9), bases (12), and others (3).

**Figure 2:**
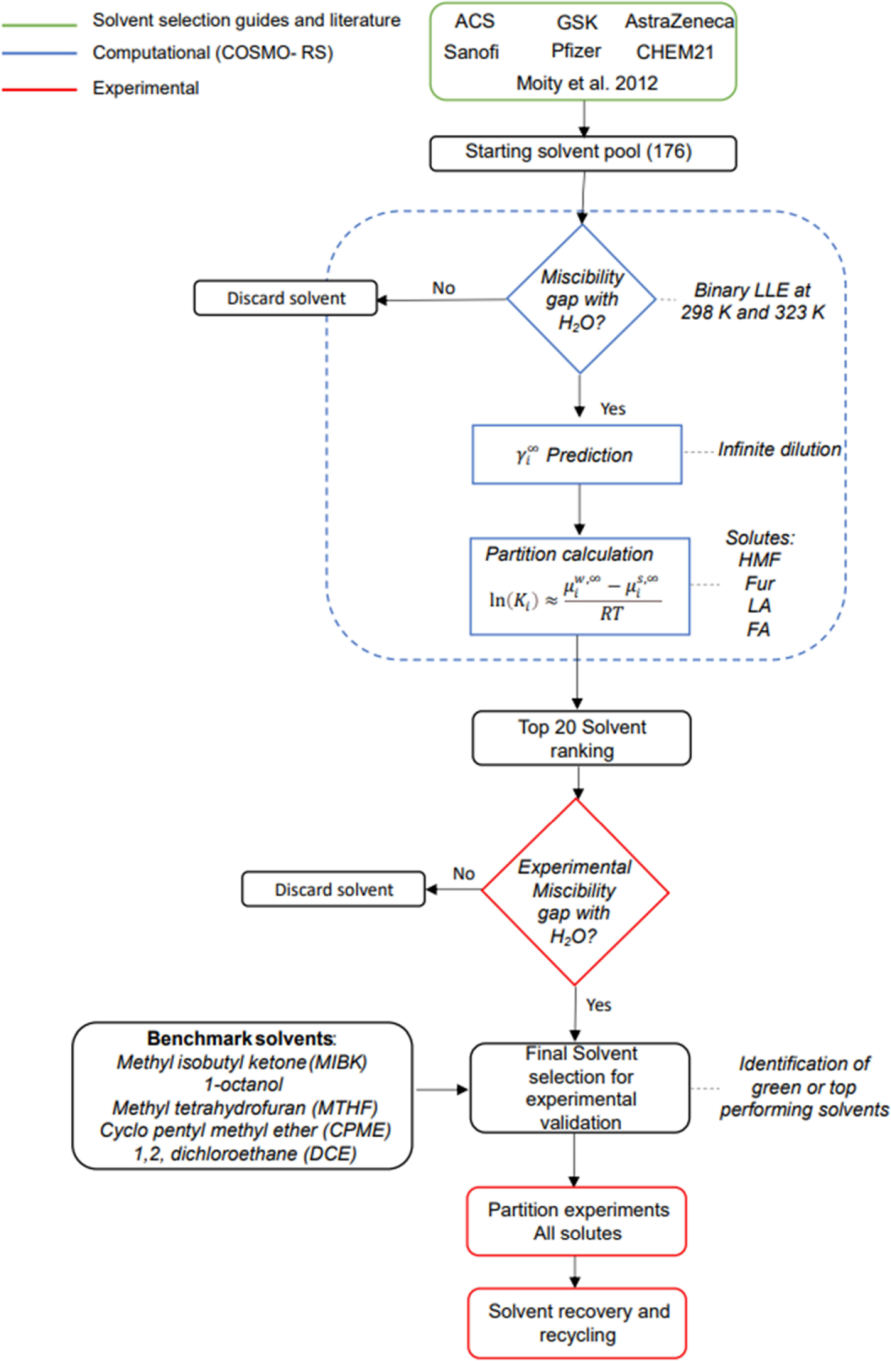
Workflow implemented to identify solvents suitable for HMF and Fur extraction using computationally guided tools (COSMO-RS) and subsequent experimental validation of partitioning and finally solvent recovery and recycling for HMF and Fur extraction.

Here, the COSMO-RS method is implemented practically through the use of COSMOthermX version 18.0.2 release 29.08.18.^12,46^ All selected solvents and solutes are included in the extended database available, COSMObase V20, with BP_TZVP_18 parametrization. Initial screening of solvents involved the identification of a miscibility gap, such that a biphasic system is observed between the solvent and water. This miscibility gap was determined through the calculation of binary liquid–liquid equilibria (LLE), at 298 and 323 K, using the COSMO-RS method. Solvents with an identified miscibility gap were then considered for evaluation of extractive capability through partition coefficient (*K*_*i*_). This can be approximated using the values of the activity coefficients at infinite dilution in binary LLE predicted by COSMO-RS, eq 1(47−49):
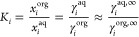
1where subscript *i* refers to the solute, superscript aq and org are the aqueous and organic phases, respectively, *x* the mole fraction, γ the activity coefficient, and γ_i_^∞^ the activity coefficient of a solute at infinite dilution. Thus, the activity coefficient of the solute needs to be calculated using chemical potentials (μ) as per eq 2:
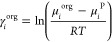
2where superscript *P* is the pure substance *i*. Finally, the logarithmic partition coefficients and hence partitioning of a solute in a biphasic system can be calculated via eq 3.
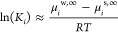
3Here, μ_*i*_^w,∞^ refers to the chemical potential of the solute at infinite dilution in water, and μ_*i*_^s,∞^ is the chemical potential of the solute at infinite dilution in the solvent. The workflow followed for this study is presented in Figure 2, wherein the steps are visualized, encompassing both the computational approach, solvent selection, and experimental validation.

### Hansen Solubility Parameters

The likelihood of solute solubility (HMF, Fur, LA and FA) in the 11 solvents to be experimentally validated was assessed via the calculation of HSP using HSPiP software (Version 5.0 m, UK).^50^ Compounds not included in the HSPiP database were constructed using the SMILES code with the Yamamoto–molecular break method and the DIY tool.^51^ The HSP consists of three parameters, dispersion (*δ*_*D*_), dipole moment (*δ*_*P*_), and hydrogen bond (*δ*_*H*_) interactions, which are then used to calculate the solubility of a given solute in a solvent through the determination of the relative distance (*R*_*a*_) between the solute and solvent in the so-called Hansen solubility space, as demonstrated in eq 4:
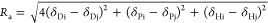
4where δ_*Di*_ and δ_*Dj*_ represent HSP for solute *i* and solvent *j,* respectively. Furthermore, HSP can be used to calculate the relative energy difference (RED, eq 5):
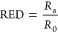
5The RED provides a method for evaluating the probability of solute dissolution in a given solvent, with RED < 1 representing a high probability of dissolution, RED = 1 a moderate chance of dissolution, RED > 1 a lower probability, and RED = 0 being a perfect system where the solute perfectly dissolves.^52^

Here, *R*_*0*_ is the interaction radius and *R*_*a*_ is the distance between the solute and solvent. *R*_*0*_ and RED were calculated with the application of the Classic Hansen technique. Initially, the scores of ten representative recommended solvents, namely, acetone, anisole, ethanol, ethyl acetate, IPA, methanol, methyl ethyl ketone, *n*-butyl alcohol, *tert*-amyl methyl ether, and water, were computed using their respective activity coefficients at infinite dilution, *ln(γ)*, generated by COSMO-RS and parametrized from 1 to 6, as suggested by Abbott and Hansen.^53^ With *ln(γ)*, lower values (scores) represent higher potential solubility. Then, a score of 1 indicates the best solvent and a score of 6 indicates the worst. After considering these scores, it was possible to refine the model and allow for fitting and calculating the *R*_*0*_ and RED of the selected solvents.

### Partition Experiments

Partition experiments were performed to determine the distribution of solutes across the aqueous biphasic system. This system comprised an aqueous and an organic phase in a 1:2 ratio by volume (typically 3 to 6 mL) held in a 15 mL falcon tube, to which 1 wt % of the solute (30 mg) with respect to aqueous phase mass is added to approximate conditions of infinite dilution.^3,18,47,54^ The solute mass was weighed using a Mettler Toledo NewClassic MS (±0.0001 g) balance. The pairs consisting of water and the organic solvent were presaturated overnight to ensure constant volumes of both phases during the extraction process. The falcon tubes were subjected to vigorous stirring at 298 and 323 K (343 K for the particular case of 4-isopropylphenol) at 900 rpm for 3 h using a Labnet Vortemp 1550, to ensure complete mass transfer. Subsequently, the samples were centrifuged with a Labnet Spectrafuge at 6500 rpm to ensure complete phase separation and a clear phase boundary. Finally, the samples were left for 16 h in a Labnet Accublock dry bath at a fixed temperature to ensure complete phase equilibria. All partition experiments were performed in triplicate, and eq 6 was used to calculate the partition coefficient^14^:
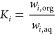
6where *K*_*i*_ is the ratio of mass fraction (*w*) of the solute (*i*) in the organic (org) and the aqueous (aq) phase of a biphasic system.

### Solvent Recovery and Performance over Reuse

Solvent recovery and reuse was assessed for the highest performing solvents for HMF and Fur alongside the reference solvent, MIBK. The volume ratios and concentration of the solute were kept the same as in the partition experiments, although the volumes were scaled to 20 and 40 mL of the aqueous and organic phase, respectively. These experiments were conducted in 100 mL round-bottom flasks at a constant temperature of 323 K, at 900 rpm for 3 h, on a RadleyCore+ hot plate with a heat-on dry block attachment. Then, the flask is left at 323 K without stirring for 16 h to ensure phase separation and split with a separating funnel. The organic phase and solute were then separated under vacuum with the use of an IKA RV10control rotary evaporator, with an IKA HB10 oil bath and recirculation of a refrigerant supplied by a Lauda MC600 MicroCool. The recovered solvent is then returned to a round-bottom flask and another aqueous phase is added, with the ratio of 2:1 organic to aqueous ratio observed and 1 wt % of solute. This was repeated for three reuse runs in total in addition to the initial extraction.

### Sample Analysis

Sample analysis for each phase was performed using an HPLC device (Agilent 1260 Infinity) with a quaternary pump (G1311B), a G1367E HiP ALS autosampler, a G136A TCC column oven, a G1315D VL diode array detector (DAD), and a G1362A refractive index detector (RID). Dilution of the organic phase with IPA was required in a ratio of 1:10:10, sample to IPA and water by volume. A Bio-Rad Aminex HPX-87H (300 mm × 7.8 mm) column was used at 65 °C, using wavelengths of 277 nm (Fur) and 282 nm (HMF) for the DAD and detector temperature of 55 °C for RID (LA and FA detection), respectively. A mobile phase of aqueous trifluoroacetic acid (0.05% v/v) was applied at 0.6 mL.min^–1^, for a duration of 60 min. Calibration curves for the HPLC are provided in the SI for HMF, Fur, LA, and FA in Figure S1.

## Results

### Solvent Screening by COSMO-RS Predictions

**Figure 3:**
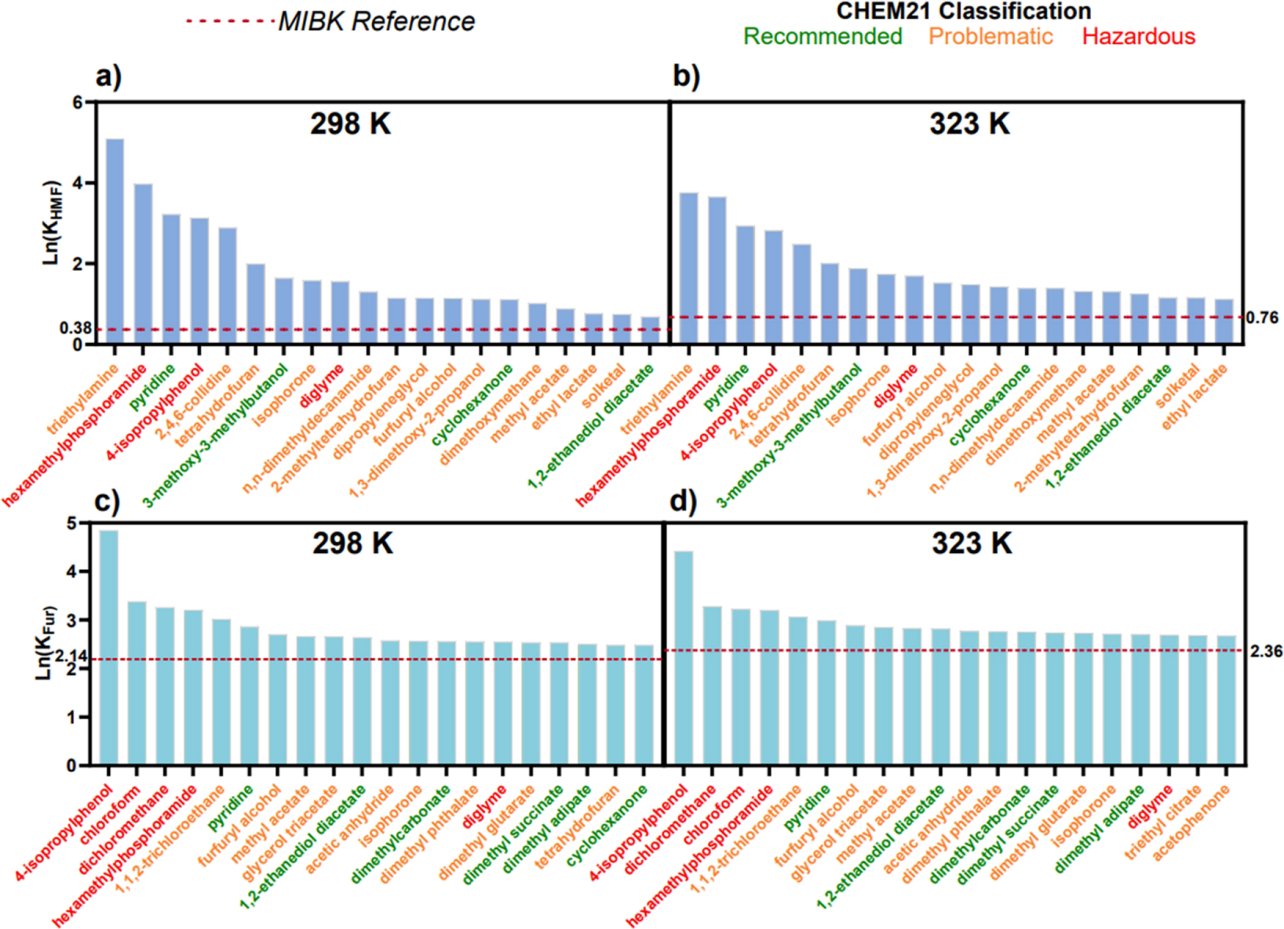
COSMO-RS-based predictions of the partition coefficient for the top 20 solvent candidates for biphasic furan extraction: a) HMF at 298 K, b) HMF at 323 K, c) Fur at 298 K, and d) Fur at 323 K.

Through estimation of binary LLE, COSMO-RS predicted the occurrence of a miscibility gap for 132 and 137 solvents at 298 and 323 K, respectively. The full list of solvents screened is provided in Table S2, which was constructed with common solvents in industrial practice as well as biobased alternatives with an increasing interest in use.^3,16,18,47^ Following initial miscibility screenings, chemical potentials of both HMF and Fur at 298 and 323 K were predicted with COSMO-RS and subsequent partition coefficients calculated using eq 3. The top 20 extraction solvents, ranked by partition coefficient, for biphasic extraction of HMF and Fur are presented in Figure 3. These rankings also include the use of a reference solvent, MIBK in this case, as a benchmark for comparison due to its widespread use as an extraction medium for HMF and Fur synthesis in the academic open research literature.^3,18^ These MIBK partition values were calculated using the same methodology as the other solvents in top 20. In addition, the CHEM21 solvent selection guide was implemented to assess and screen the candidates on the basis of their EHS profile; thanks to the spreadsheet tool supplied in the CHEM21 solvent selection guide.^16^ This guide allows classification into different categories, namely, recommended, problematic and hazardous denoted green, orange, and red, respectively, as featured in Figure 3. The full list of calculated EHS parameters is available in Table S3.

The temperatures chosen for the partition experiments are 298 K as a reference temperature and 323 K to allow evaluation of temperature effects without approaching reaction temperatures of c. 373 K.^8^ Starting with the ranking for HMF at 298 and 323 K, the top 20 candidates identified show higher extractive capability than the MIBK reference. The top nine solvents at both temperatures studied are identical, only differing in partition magnitude with a general trend of lower partition values at a higher temperature. However, limitations with the semiquantitative nature of COSMO-RS persist, with certain solvents predicted to show a miscibility gap with water yet the opposite is true in reality. Four solvents have hence been removed for their miscibility with water and disregarded for further work, namely, hexamethylphosphoramide, 3-methoxy-3-methyl-butanol, tetrahydrofuran, and solketal, with the miscibility having been experimentally confirmed. These omissions leave triethylamine as the extraction solvent with the highest predicted partition coefficient. The same solvents were omitted from the Fur extraction ranking where relevant, with the final top-ranked solvent for Fur extraction deemed to be 4-isopropylphenol. These rankings, alongside guidance from the CHEM21 solvent guide and exploration of other commonly used extraction solvents, have led to a final set of 11 solvents for subsequent experimental studies. These solvents include the top candidates for both HMF and Fur, triethylamine and 4-isopropylphenol, and other high-performing candidates that were classed as either problematic or recommended, isophorone, cyclohexanone, 2-methyltetrahydrofuran (MTHF), dimethyl carbonate (DMC), and 1,2-ethanediol diacetate. Three additional solvents were considered as further special interest out of the top 20 predictions, namely, 1-octanol, cyclopentyl methyl ether (CPME), and 1,2-dichloroethane (DCE) owing to them being reference solvents. 1-octanol partitioning is commonly used to assess a solute partition in environmental chemistry or toxicology,^55^ whereas CPME for use in the extraction of biobased compounds like phenolics and can be derived through Fur, an example of a circular economy,^56^ and DCE for HMF-analogue extraction, respectively.^57^ Extra relevance of these two final solvents can be attributed to applications to wider one-pot production Furans from lignocellulosic biomass, wherein phenolic compounds found in lignin may be extracted or HMF synthetically upgraded to an analogue such as 5-chloromethylfurfural or 5-bromomethylfurfural.^58^ Previous work has reported the extraction of HMF in aqueous biphasic systems with MIBK, CPME, MTHF, cyclohexanone, and DMC at temperatures between 298.15 and 323.15 K in concentrations of the solute in the aqueous phase from 0.64 to 1.5 wt %.^15,59−62^ When Fur is the solute, the solvents tested were MIBK, CPME, MTHF, cyclohexanone, and isophorone also between 298.15 and 323.15 K from 0.8 to 1.71 wt %.^17,63−65^ With the shortlisting of candidates proposed, this work will provide new experimental partitioning data for 1-octanol, DCE, and 1,2-ethanol diacetate for both solutes in addition to isophorone for HMF and DMC for Fur.

Furthermore, knowledge of mutual solubilities of components in a biphasic system is required to identify and minimize water sorption effects and evaluate the capability of solvent–water or water–solvent leaching that occurs throughout the process. Table S4 presents the solvent in water and water in solvent solubility for the nine solvents studied at 298 and 323 K. A range of mutual solubilities exists for solvent in water from 0.0005 g.ml^–1^ (1-octanol) to 0.1600 g.ml^–1^ at 298 K (1,2-ethanediol diacetate).^66,67^ The reference solvent MIBK lies approximately in the middle of these solubility values,^68^ with one of the lowest values of water in solvent solubility at 0.0155 g.ml^–1^, thus exhibiting minimal water leaching into the organic layer. This effect is 10-fold higher in CPME than MIBK, despite the relatively similar solvent in water solubilities, highlighting the importance of knowledge of both sets of mutual solubilities.

## Experimental Results

### Partitioning of Furans in Aqueous Biphasic Systems

**Figure 4:**
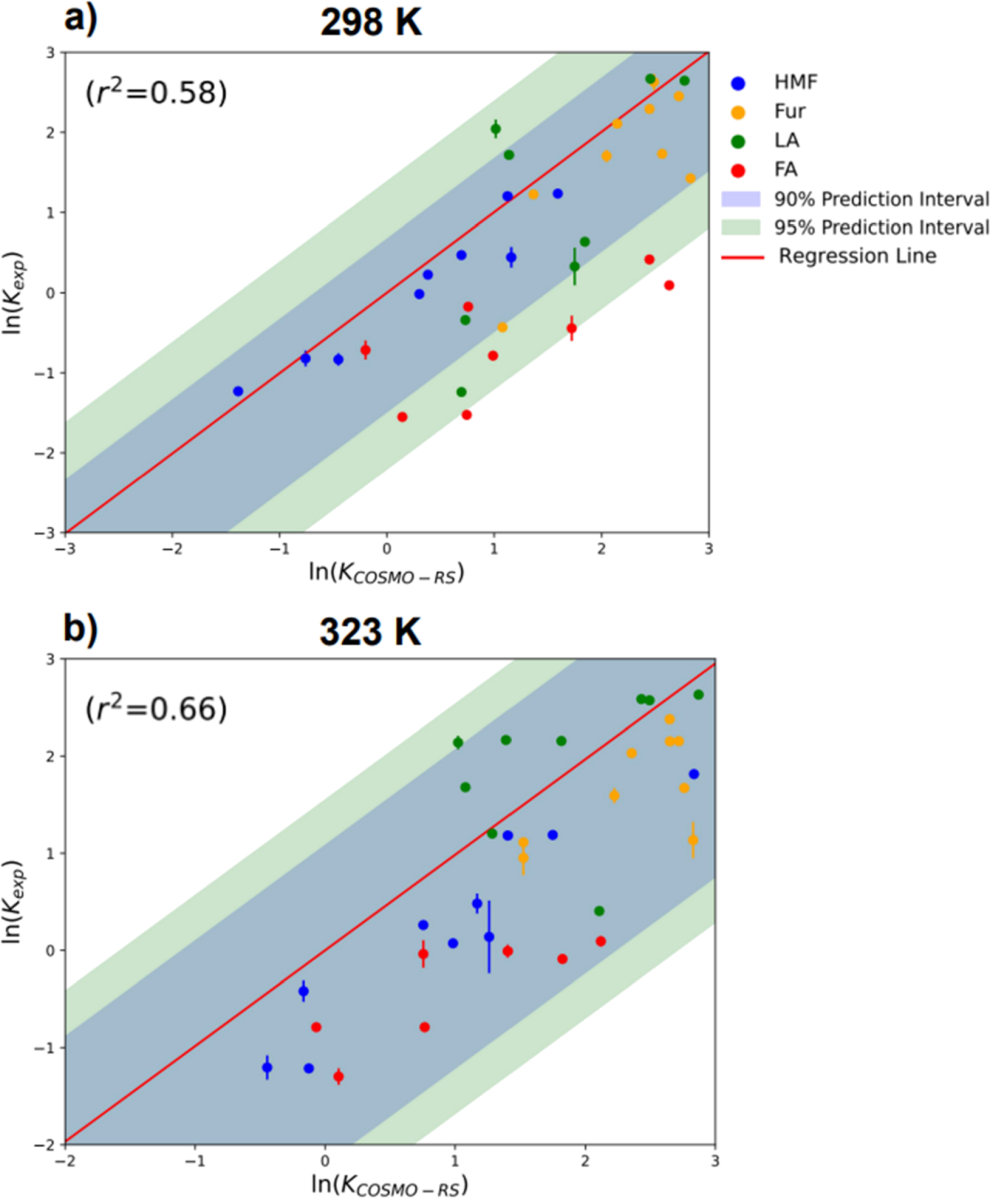
Comparison between experimentally determined partition coefficients and those predicted by COSMO-RS for four solutes, HMF, Fur, LA, and FA at (a) 298 K and (b) 323 K.

The partitioning of HMF and Fur in aqueous biphasic systems was investigated at 298 and 323 K. In addition, LA and FA were also assessed given their presence in the reaction network involved in the production of HMF, as shown in Figure 1. These experimentally determined partition coefficients were then compared to those predicted using the COSMO-RS method, providing moderate levels of fit with regression coefficients of 0.58 and 0.66 at 298 and 323 K, as shown in Figure 4a,b. Individual comparisons of partition coefficients for each solvent and solute can be found in Figures S2 and S3, where the root mean square error (RMSE, eq 7) for each set of solvent–solute combination is shown.
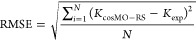
7

The comparisons revolved around the calculation of simple linear regression with respect to experimentally determined partition coefficients and those predicted by COSMO-RS. Additionally, the confidence intervals were calculated such that the respective amount of data fit within 90% and 95% of these bounds. Figure 4a details the comparison of the whole set of studied solvents and solutes at 298 K, whereas the individual RMSE for HMF, Fur, LA, and FA are 0.76, 5.23, 3.68, and 0.68 are presented, respectively in Figure S2. The general trend for HMF indicates a minor overprediction by COSMO-RS of the partition values; this low RMSE indicates minor deviation and a positive correlation between estimated parameters and experimental values.

According to the experimental results, the highest logarithmic partition coefficient at 298 K was achieved by isophorone, at 1.07 (*K*_*HMF*_ = 2.93) for HMF. This cyclic ketone’s COSMO-RS-predicted partition value is higher than the experimental value at 1.59, suggesting overestimation. Three other solvents, MTHF, 1,2-ethanediol diacetate, and cyclohexanone at logarithmic partition coefficient values of 0.44 (*K*_*HM*F_ = 1.56), 0.47 (*K*_*HMF*_ = 1.60), and 0.70 (*K*_*HMF*_ = 0.70), respectively, experimentally showed higher partition coefficients than MIBK. Furthermore, the value of ln (*K*_*HMF*_) using cyclohexanone at 0.70 (*K*_*HMF*_ = 2.02) is relatively similar to that presented in the literature under similar conditions at 0.99 (*K*_*HMF*_ = 2.69).^15^ These results for MIBK partitioning are in line with those presented in the literature, with results between 0.00 (*K*_*HMF*_ = 1.00) and 0.69 (*K*_*HMF*_ = 1.99) at 120–220 °C.^69−71^ Although experimental results here suggest a poor extractive capability, successful use of 1-octanol for HMF extraction from an aqueous biphasic system was demonstrated by Zhao et al., in which 1-octanol showed the high stability with HMF due to the hydrogen bonds formed.^57^ DMC in this work was observed to have a logarithmic partition coefficient of −0.06 (*K*_*HMF*_ = 0.94), which indicates unfavorable partitioning for the purpose of separation from the aqueous phase. However, success has been reported by numerous works where the value of DMC as a green bioderived solvent is identified, with high stability and little interference with the HMF molecule, although partition coefficients were not presented in the work.^72^ Sayed et al. presented a logarithmic partition coefficient of 0.18 (*K*_*HMF*_ = 1.20) for DMC at 295 K, comparable to those experimentally determined in this work with a continuous production method of fructose dehydration toward HMF.^62^ CPME is observed to have poor partitioning in this work at a value of −0.83 (*K*_*HMF*_ = 0.44), which is consistent with findings reported by Zilnik et al. at low wt % of HMF loading in (<1%).^60^ A notable point to make is the omission of triethylamine from any of the experimental results. This removal was due to the observed color change of the solutions after the partitioning experiments, indicating that the reactions between the solvent and HMF and Fur occurred (Figure S4).

Fur partitioning predictions are less in line with experimental values than those for HMF, with a higher RMSE value of 5.23, as seen in Figure S1b. As a whole, the partitioning of Fur is greater than that of HMF due to the higher inherent hydrophobicity resulting from the lack of the −OH moiety. The highest value of *ln*(*K*_*Fur*_*)* was deemed to be with cyclohexanone at 2.6 (*K*_*Fur*_ = 13.45) with isophorone a close second at 2.45 (*K*_*Fur*_ = 11.58). Isophorone has seen success when implemented as an extraction phase for biphasic Fur production from xylose and birch hydrolysate.^73^ Additionally, Ershova et al. provides robust LLE quantification of Fur extraction using isophorone at 303 K, with *ln*(*K*_*Fur*_*)=2.63* (*K*_*Fur*_ = 13.89) at 0.8 wt % Fur in the aqueous phase.^65^ cyclohexanone is the only solvent evaluated with a higher partition coefficient experimentally than those predicted through the COSMO-RS method. In comparison to MIBK, a total of three solvents outperformed this reference solvent, isophorone, cyclohexanone and DCE. Finally, following the CHEM21 classification of these solvents, it would be recommended to use either cyclohexanone or isophorone as the extraction solvent with these ranked as recommended and problematic, respectively, when compared to hazardous DCE.

Knowledge of both LA and FA partitioning in these biphasic systems is relevant considering their concomitant generation as byproducts in the production of HMF, owing to its potential rehydration. Furthermore, LA is a platform chemical in its own right with numerous works detailing the potential synthetic upgrading pathways.^74^ The extraction of LA yields results of RMSE of 3.68 with numerous solvent partitioning under and over-predicted by the COSMO-RS method, as shown in Figure S3c. A total of three solvents, CPME, cyclohexanone, and DMC provided higher experimental values of partitioning. One of the highest performing solvents was identified as MTHF with a *ln*(*K*_*LA*_*)* of 2.65 (*K*_*LA*_ = 14.15); however, MTHF is generally not considered as a solvent for LA extraction in the literature as MTHF is a commonly produced derivative of LA through hydrogenation.^75^ Similar to HMF and Fur extraction, isophorone and cyclohexanone were identified as the best performing solvents, with a *ln*(*K*_*LA*_*)* of 2.73 (*K*_*LA*_ = 15.33) and 2.69 (*K*_*LA*_ = 14.73), respectively. cyclohexanone, with its recommended ranking in the CHEM21 solvent selection guide coupled with the high extraction, proves to be a promising candidate for extraction in this scenario.^76^ Generally, COSMO-RS predictions for the partitioning of FA are in line with validation attempts, with an RMSE of 0.68. The values of *ln*(*K*_*FA*_) are generally lower than those exhibited by HMF and Fur due to the increased polarity of the FA molecule. The highest experimentally determined partition coefficient was MTHF at a value of 0.42 (*K*_*FA*_ = 1.52), with the next highest, isophorone at 0.09 (*K*_*FA*_ = 1.09).

Isolated production of each of the compounds analyzed is rarely achieved in real systems, especially for the production of HMF where rehydration products, LA and FA, are formed. Hence, it is important to consider the selectivity of solvents toward desired products to maximize the leverage of biphasic systems. A simple measure of selectivity can be represented by a ratio of partition coefficients (eq 8).
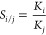
8where *i* represents the desired product and *j* the undesired products. This can also allow for the quantification of Fur selectivity over HMF selectivity in systems where simultaneous production can take place, such as those utilizing complex biomass with both hexose and pentose sugars.

**Figure 5:**
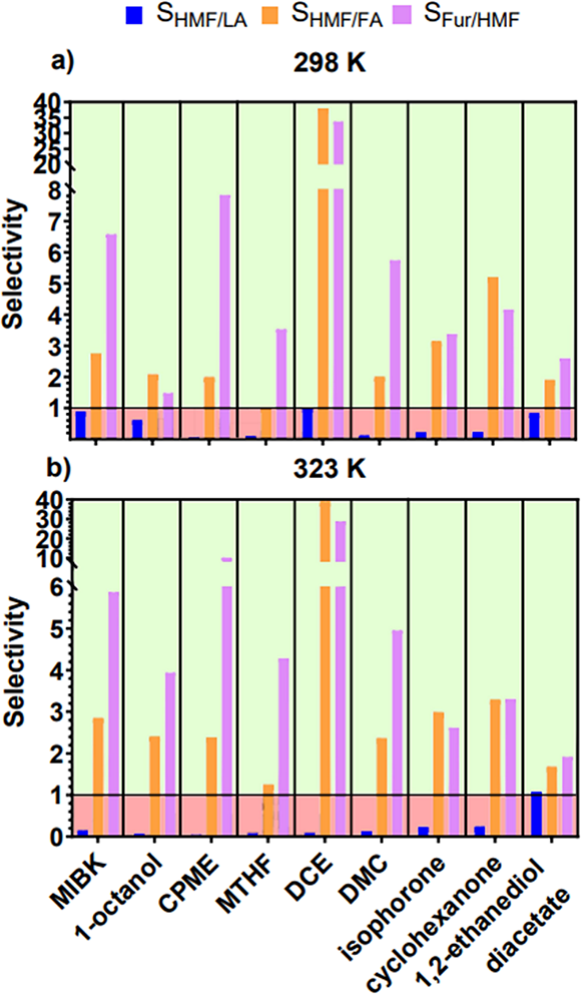
Selectivity of each solvent toward LA and FA with respect to HMF and HMF with respect to Fur for extraction at (a) 298 K and (b) 323 K. The green shaded region indicates preferential selectivity toward HMF in comparison to LA or FA, or selectivity toward Fur in comparison to HMF, with the red region representing the opposite.

Figure 5a,b show the calculated selectivity at 298 and 323 K, respectively, providing insights into solvents in which preferential extraction would occur. Every solvent has a significantly higher selectivity toward HMF than with FA except for MTHF at 298 K. This high selectivity toward HMF over FA can be attributed to the difference in polarity between the two molecules. Conversely, the relative nonpolarity of LA results in a selectivity below unity for all studied solvents except for 1,2-ethanediol diacetate at 323 K. Furthermore, when considering potential simultaneous production of HMF and Fur, analyzing selectivity toward each furan can also be helpful as this will happen when converting complex biomass. In this case, Figure 5 also represents the selectivity of the partitioning of Fur to HMF. All considered solvents observe a selectivity greater than unity, indicating clear preference toward the former. In general, the COSMO-RS predictions of partition coefficients increased with respect to the temperature, aside from the values generated for FA, which decreased marginally, as seen in Figure 4b. However, this increase also correlates to the increase in RMSE of all solutes, except FA which decreased from 0.68 to 0.50. Finally, the linear regression (Figure 4b) increases in line with the increase in temperature to a value of 0.66. Highlighting the individual extraction solvent such as the substantially increased *ln(K*_*HMF*_*)* of cyclohexanone from 0.70 (*K*_*HMF*_*= 2.02*) at 298 K to 1.18 (*K*_*HMF*_*=* 3.26) at 323 K allows for informed choices for use of extraction solvents in biphasic reaction systems. Additionally, cyclohexanone was the second most effective extraction solvent identified at 323 K, marginally lower than isophorone. The highest performing extraction solvent, for both HMF and Fur, was 4-isopropylphenol, as seen in Table S5. Instead of 323 K, these partition coefficients were determined at 343 K as the melting point of 4-isopropylphenol is 334 K.^77^ The increase in partitioning of HMF with respect to temperature was fairly marginal across all investigated solvents, with only MTHF and DMC decreasing in capacity. Fur partitioning at 323 K on the other hand reported values of greater than zero for all solvents studied. However, the standout exemplar extraction solvents, cyclohexanone, isophorone, and DCE, all decreased in *ln*(*K*_*Fur*_*).* The most significant of these three solvents that decreases in capacity was isophorone. The decrease in capacity for DCE is less severe, but with the focus on green chemistry in this work, the choice to investigate an identified hazardous solvent was declined for further work involving solvent recovery and recycle. The influence of temperature on LA extraction is the most significant out of all the solutes studied, with the majority of solvents reporting a significant increase in the values of partition coefficient. The greatest increase was observed with 1-octanol where an initial *ln(K*_*LA*_*)* of −0.34 (*K*_*LA*_ = 0.71) at 298 K was reported, which increased to 2.14 (*K*_*LA*_ = 8.50) at 323 K. Interestingly, in the case of FA, only a single partition coefficient determined experimentally is greater than zero, namely, isophorone at 0.09 (*K*_*FA*_ = 1.09). Generally, the COSMO-RS predicted values for FA lie relatively close to the parity line, with a slight tendency for overestimation of partition coefficients. The poor extraction of FA relative to both HMF and LA occurs as a result of molecular polarity, thus generally unsuitable for dissolution on nonpolar extraction solvents.

### Comparison of COSMO-RS Predictions and Partitioning of HMF and Fur with Previous Work

**Figure 6:**
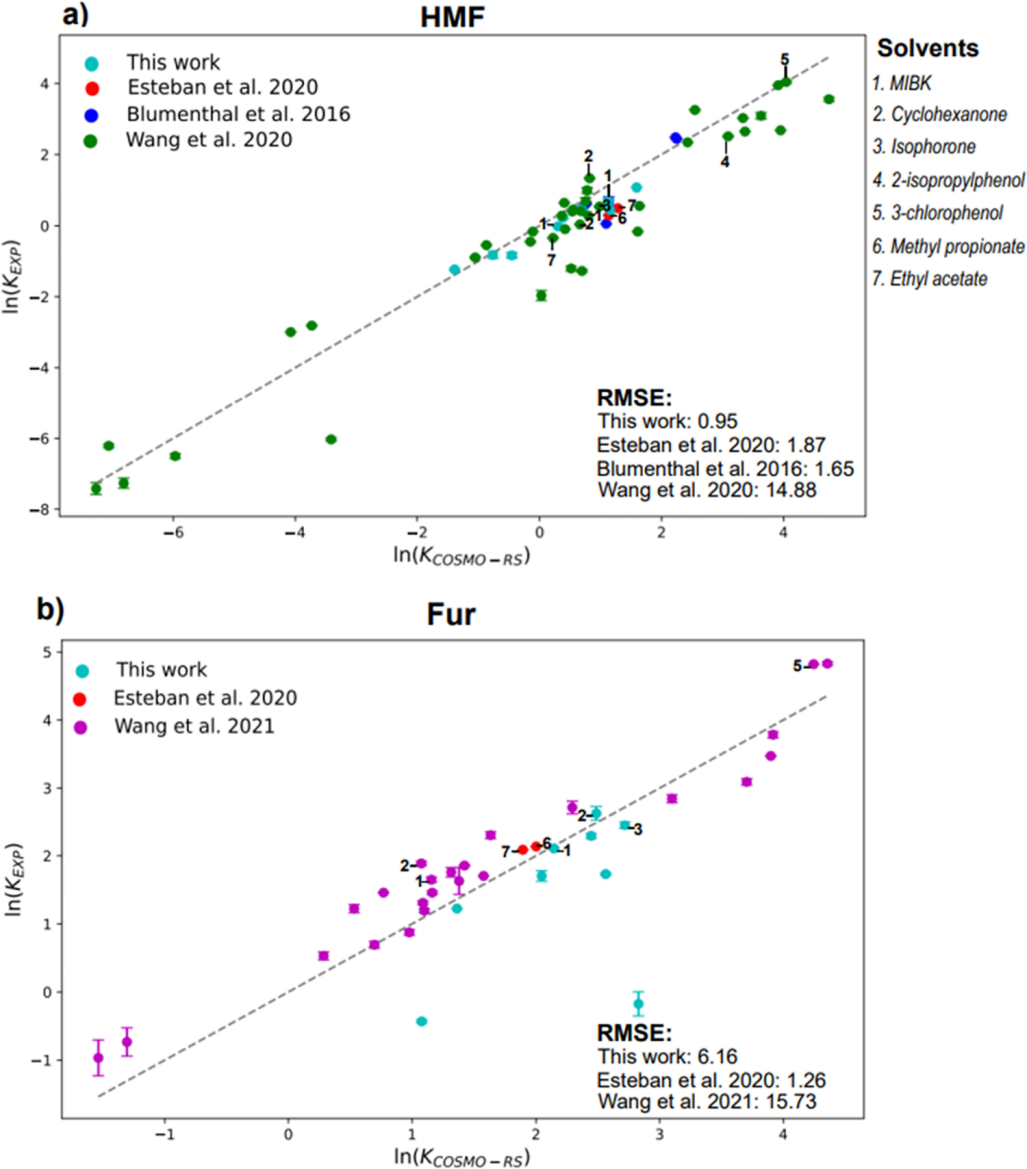
Comparison of partition coefficients of (a) HMF and (b) Fur in different solvents in this work and those from the literature to COSMO-RS predicted values at 298 K with 1 wt % solute in the aqueous phase. Note: for Blumenthal et al. which was 1 wt % equivalent of whole solutions and Esteban et al. which was 0.7 wt % of aqueous phase for (a) HMF^14,15,18^ and (b) Fur.^17,18^ Additionally, common solvents between works or those that were highest performing were labeled, 1 through 7.

Previous large scale studies investigated COSMO-RS solvent screenings for HMF and Fur partitioning in aqueous biphasic media employing molecular solvents with the corresponding experimental validation.^14,15,17,18^ At 298 K, Figure 6a provides the parity plot for HMF partitioning in relevant studies and this work, showing a lower deviation than others (RMSE = 0.95). The variation in RMSE can be attributed to variations in determination of both the experimentally validated partition coefficients and the COSMO-RS predicted values. The former deviates from partition experiments in this article with a slightly different concentration of solute with a 1 wt % of the total solution used instead of only the aqueous phase. The COSMO-RS prediction methodology remains similar to the use of infinite dilution assumption to calculate the partition coefficient if a binary LLE was observed. The highest RMSE was generated by Wang et al. at a value of 14.88, although this work includes significantly more data points in the series analyzed with 39 separate data points.^14,15^ Solvents with the high experimentally determined partition coefficients have been identified in Figure 6, with the highest in all data sets provided as 3-chlorophenol for HMF and Fur, although it is worth mentioning that this halogenated phenolic compound is considered as hazardous by CHEM21.^16^ As such, it would constitute a questionable recommendation for use in a process that aims to be green. As expected, the partitioning of Fur is greater than HMF in the literature due to the increased nonpolarity of Fur through lack of the −OH moiety. Other identified solvents include methyl propionate and ethyl acetate by Esteban et al; however, these would be generally unsuitable for reaction systems due to the hydrolysis provided by these esters.^3,18^ Additionally, there exists numerous similarities between values of *K*_*HMF*_ reported in the literature and, in this work, with values of 1.06, 1.04, and 1.25, respectively.^14,15^

Figure 6b details the literature that reports experimental and COSMO-RS predicted *K*_*Fur*_ values at 298 K under 1 wt % of Fur in the aqueous phase, except for 0.7 wt % for Esteban et al.^3,18^ These values of partitioning are higher in general than those for HMF as expected, with the maximum experimental value in the data set of 125 achieved with 3-chlorophenol. Data points are significantly more distributed along the parity line as described by the increased RMSE values over the HMF data set. Additionally, the RMSE in this work can be attributed to some significant deviations and model overestimation such as 1,2-ethanediol diacetate with *K*_*EXP*_ of 0.84 and *K*_*COSMO-RS*_ of 16.93. In general, this work reports higher experimental partition values than those presented in the literature. The deviation between our presented COSMO-RS values and those presented in Wang et al. relates to the different computational and DFT basis implemented in the initial solvent screen.^15,17^

### Thermodynamic Insights into Solute–Solvent Interactions

#### Sigma Profiles

**Figure 7:**
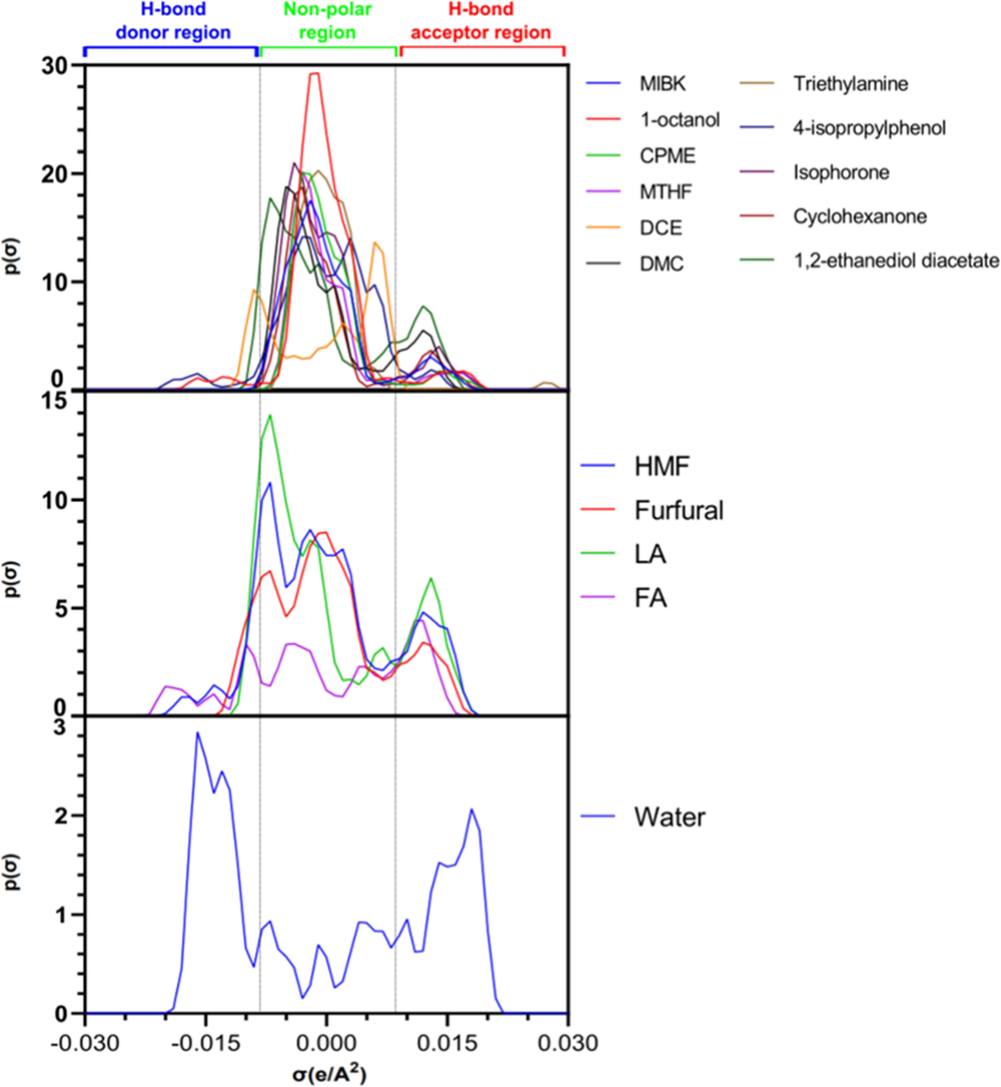
Sigma profiles generated by the COSMO-RS method for lowest energy conformers for the solutes and solvents studied in this work.

As an essential part of the calculations and discussion around the COSMO-RS method, the σ-profiles can be split into three distinct regions, namely, hydrogen bond donor (HBD) at values of σ < – 0.0082 e Å^–2^; nonpolar at −0.0082 e Å^–2^ ≤ σ ≤ 0.0082 e Å^–2^, and hydrogen bond acceptor (HBA), at σ > 0.0082 e Å^–2^.^11,78^Figure 7 displays these probabilistic charge distributions for the four studied solutes and solvents, where these distributions can be used to infer interactions between compounds. First, looking at the σ-profile generated for HMF, it is evident that three large distinct peaks and a lesser peak are observed. The first two large peaks are present in the nonpolar region and one in the HBA region at values of −0.007, – 0.002, and 0.012 e Å^–2^, respectively. This peak in the HBA region, which is also present in Fur, can be attributed to the carbonyl groups present, with LA and FA with carboxylic groups present. The range of solvents studied has a myriad of σ-profiles that can be mapped onto the solute profiles where again the like dissolves like. A standout solvent in terms of the magnitude of charge distribution is 1-octanol in the nonpolar region as a result of its hydrocarbon chain. The highest predicted extraction solvent for Fur is 4-isopropylphenol with small slight peaks in both the HBA and HBD regions, corresponding to the hydroxyl moiety of the molecule, thus showing the resemblance of the charge distribution of both compounds. For HMF, triethylamine is the best predicted extraction solvent by the COSMO-RS method, although the shape of the σ-profile generated is somewhat different to that of HMF. These σ-profiles provide general indication of favorability of solubility of compounds wherein like dissolves like, although the absolute magnitudes are related to the molecular size primarily.

#### Thermodynamic Analysis Using COSMO-RS

**Figure 8:**
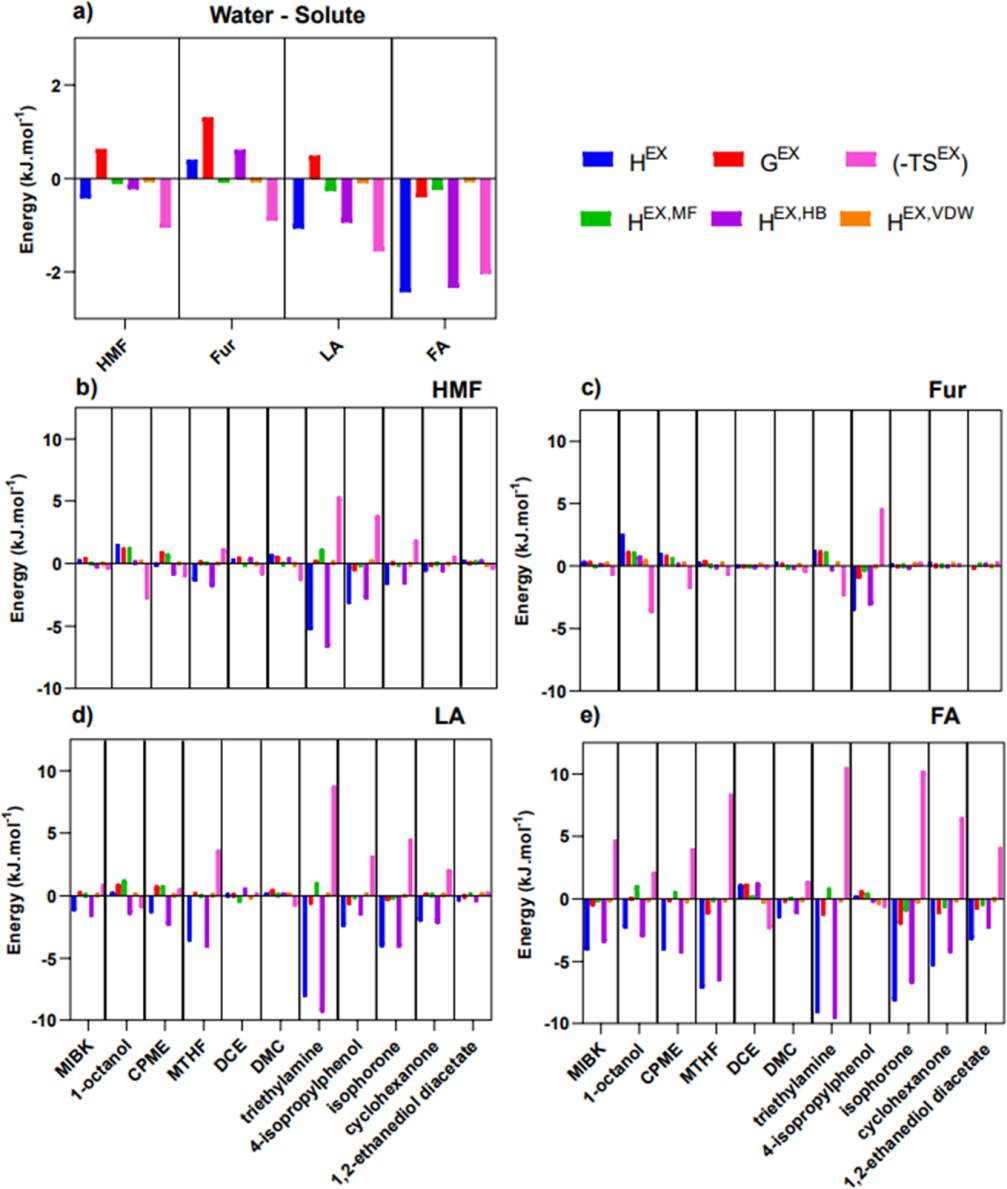
COSMO-RS calculations of the excess enthalpy (H^EX^), with three constituent parts MF – misfit forces, HB- hydrogen bonding, VDW- Van Der Waals, excess free energy (G^EX^), and entropy (-TS^EX^) at 298 K for binary mixtures of (a) water-HMF, Fur, LA, and FA, (b) HMF-solvent, (c) Fur-solvent, (d) LA-solvent, and (e) FA-solvent.

COSMO-RS provides further insights into the behavior of solute–solvent molecular interactions through the calculation of excess properties and energetic contributions. In particular, the excess Gibbs free energy (*G*^EX^) and the excess enthalpy (*H*^EX^) were calculated with the COSMO-RS method, while the term related to the excess entropy (−*TS*^EX^) was calculated using eq 9:
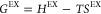
9Moreover, *H*^EX^ can be seen as the sum of three energetic contributions (i.e., *H*^EX,*i*^), where *HB* denotes hydrogen bonding, VDW refers to van der Waals forces, and MF represents electrostatic interactions as per eq 10:
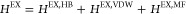
10It is worth noting that, besides HMF and Fur, the computational study was expanded to include the effect of the two rehydration byproducts of HMF, LA and FA, to provide insights into the behavior of trace production of these acids. These thermodynamic contributions are provided for the solute-water systems in Figure 8a and for the solute-organic solvent systems in Figure 8b–e, calculated at 298 K using COSMO-RS.

The interactions occurring in the aqueous phase (Figure 8a) provide insight into the affinity of the solutes for water, which is mainly determined by favorable hydrogen bonding in the case of HMF, LA and FA, while being unfavorable in the case of Fur; hence, the higher affinity of HMF, LA and FA for water in comparison to that of Fur can help to explain the higher extraction efficiencies experimentally obtained for the latter, which is less hydrophilic. When evaluating the interactions occurring between the solute-organic solvent systems, a higher overall tendency of HMF, LA, and FA interacts through favorable hydrogen bonding with the organic solvents studied in comparison to Fur, due to the lack of hydroxyl group in its structure.

The highest *H*^*EX*^ is observed in triethylamine for HMF at 298 K, with a large negative *H*^*EX,HB*^, which contributes to the stabilization of molecular structures of the solute–solvent interaction;^79−82^ however, the partition of HMF in aqueous biphasic systems with this solvent proved unsuccessful since a reaction was observed as discussed above. The negative *H*^*EX*^ for MTHF, triethylamine, 4-isopropylphenol, isophorone, and cyclohexanone indicates that the transfer of solute is enthalpy driven, whereas 1-octanol and CPME are entropically driven as described by the negative (−*TS*^*EX*^), albeit with relatively low values. This dominant entropic driving force for 1-octanol could be attributed to the microheterogeneous structure formed through saturation of the organic phase by water.^83^ Values of thermodynamic contributions for Fur at 298 K are in general relatively close to zero, indicating an ideal mixture, with few significant values of interest aside from 4-isoproylphenol with a negative *G*^*EX*^ and *H*^*EX,HB*^. Looking toward LA and FA, both exhibit higher values of thermodynamic contributions in general, with an order of magnitude greater when looking at Fur (with the exception of 4-isopropylphenol). The contributions of van der Waals are centered around the molecular size of the studied solute; thus, both HMF and Fur are larger than LA and FA and have higher contributions toward the excess enthalpy as expected due to the scaling of van der Waals due to molecular size. Negative values of *G*^*EX*^ infer spontaneity or a thermodynamically favorable process.^11,12,84^ Here, only select solvents display favorable processes, particularly, triethylamine and isophorone for HMF and 4-isopropylphenol for Fur.

This study was repeated for 323 K to observe the effect on the thermodynamic contributions reported here, as shown in Figure S5. The most significant of changes for the water-solute interactions were observed with the decrease in *H*^*EX*^ for all solutes, with HMF approaching an ideal mixture. Furthermore, the spontaneity of mixing expressed through the *G*^*EX*^ contributions increased by a small amount, indicating less spontaneous mixing for those with negative values, namely, FA in water. When considering the solute–solvent interactions, the majority of *H*^*EX*^ decreased with respect to temperature with only MIBK becoming negative from a positive value at 298 K. Overall, the temperature effect on thermodynamic excess contributions calculated is relatively minor and changes little in the way of the nature of driving forces (enthalpic or entropic) for the transfer within the binary mixtures.

HSP was used as a measure of prediction of solute solubility in the range of studied organic solvents. Figure S6 presents the three calculated parameters, dispersion (*δ*_*D*_), dipole moment (*δ*_*P*_), and hydrogen bond interactions (*δ*_*H*_) for HMF, Fur, LA, and FA at 298 and 323 K together with the 11 solvents assessed, where a similarity between the values of such parameters between solutes and solvents are indicative of affinity. The likelihood of dissolution between the solutes and solvents is given through the calculation of RED, eq 5. As such, RED is presented in Figure S7a,b for 298 and 323 K, respectively. The key points of interests lie in the highlighting of several solvents that were expected to perform effectively at solvent extraction, such as cyclohexanone for HMF extraction. Furthermore, this analysis was expanded to include the detailing of experimentally determined *w*_*i*,org_ with respect to RED, as shown in Figure S8a,b, for 298 and 323 K, respectively. These results highlighted that the majority of solutes at both studied temperatures showed favorable dissolution toward their respective solvents. Additionally, the majority of solvents used for the dissolution proved ineffective for extraction and this is reflected in the poor dissolution observed in tandem with the RED values >1 determined.

### Organic Solvent Recovery and Performance over Reuse

**Figure 9:**
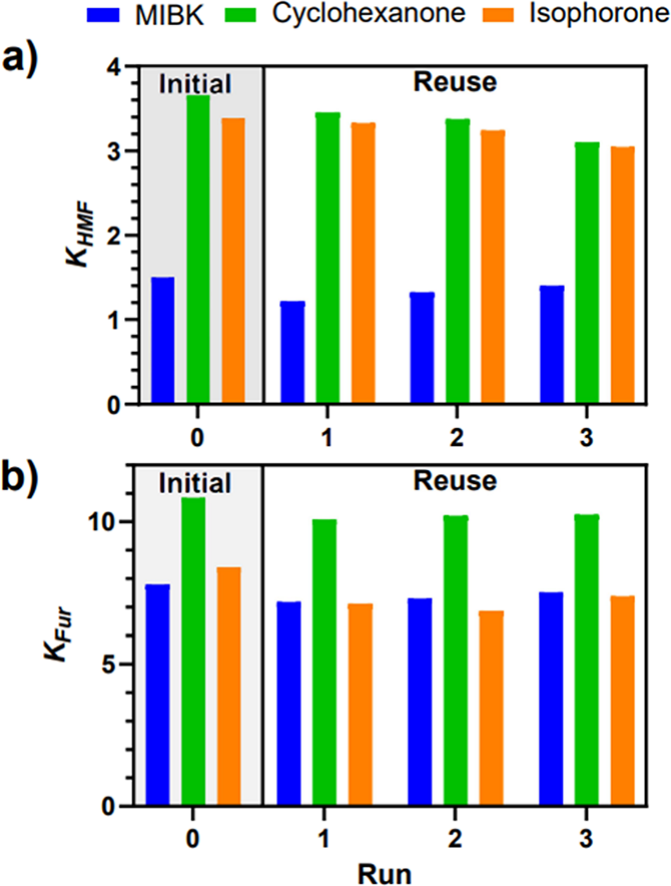
Solvent extraction performance upon recovery for an initial run and three subsequent cycles for MIBK, cyclohexanone, and isophorone at 323 K for (a) HMF and (b) Fur.

Keeping with the sustainability of the process, it is imperative to consider the stable performance of the organic solvent when conducting a liquid–liquid extraction over several reutilization cycles. Herein, we present the results of experimentally determined solvent recovery and reuse of the highest performing extraction solvents for HMF and Fur extraction, namely, cyclohexanone and isophorone, alongside, MIBK, the reference solvent. Following the calculation of the EHS parameters, isophorone qualifies as “problematic”, whereas cyclohexanone and MIBK attain a degree of “recommended”.^16^ The stability performance tests of the organic solvents comprised four runs performed at 323 K to be closer to meaningful reaction temperatures. Figure 9a presents the partition coefficient for HMF. The highest performing solvent in terms of partitioning was deemed to be cyclohexanone with a decrease in *K*_*HMF*_ of 14.25% across the entire cycle. A decline in *K*_*HMF*_ for MIBK and isophorone was observed to be 17.92% and 9.07%, respectively. cyclohexanone was observed to be the best solvent in terms of partitioning. There are additional benefits of using cyclohexanone over isophorone, with the lower boiling points of 155.6 to 215 °C, respectively.

However, lower boiling points do not directly correlate toward lower energy costs, as this is primarily driven by differences in relative volatility or the presence of azeotropes such as with water and Fur at 35.46 wt % Fur.^85^ Additionally, ASPEN plus has been used to apply the NRTL model to predict VLE of HMF and Fur with each of the three solvents to ensure no azeotropes are formed, this is presented in the SI in Figure S9. These generated VLE predictions at 101.3 kPa all show favorable differences in relative volatility, indicating that simple distillation could be utilized for separation, except in the case of cyclohexanone and Fur. The relative volatility between cyclohexanone and Fur, Figure S9f, is very low so separation through simple distillation would require large amounts of equilibrium stages to achieve high degrees of separation. Hence, process design and simulation work must be done in a broader sense to incorporate additional objectives such as energy efficiency, process performance, and sustainability in order to optimize these process conditions.

Figure 9b presents the partitioning results for Fur and the three select solvents. The highest partition coefficient is observed with cyclohexanone at 10.86 before reuse, with a 6.20% decrease across subsequent cycles. Both MIBK and isophorone exhibited excellent partitioning as expected, although the former’s decline was lesser at 6.91% opposed to the latter with 17.14%. MIBK is an excellent choice for Fur extraction, ranked as recommended in the CHEM21 guide, with excellent stable partitioning after multiple runs (<7% decline) and the relatively low boiling point of 116 °C.^16^ This low boiling point allows for either moderate vacuum distillation or even simple distillation due to the difference in volatility between Fur and MIBK, due to there being no azeotrope formation.^86^

## Conclusions

This work provides insight into combined computational (COSMO-RS) and experimental validation of selection of solvents for the extraction of HMF and Fur from an aqueous biphasic system. COSMO-RS has proven to predict acceptably the extraction performance of solvents for HMF, Fur, LA, and FA, although this method is not without limitations, such as the approximation to infinite dilution assumed in this work. The highest performing solvent for HMF extraction was isophorone at both 298 and 323 K with *(K*_*HMF*_*)* values of 2.93 and 3.28, respectively. In general, the partitioning of Fur is greater than that of HMF due to the decreased polarity resulting from the lack of the −OH moiety, additionally indicating that biphasic systems are a successful mitigation strategy for byproduct formation as emphasized with the *K*_*Fur*_ of cyclohexanone at 13.85 and 10.78 at 298 and 323 K respectively. Partitioning of LA and FA showed that the former had a great tendency for extraction in the organic phase while the latter preferentially remains in the aqueous phase bar a handful of cases, namely, MTHF at 298 K and isophorone at both 298 and 323 K. Despite showing some over and underpredictions depending on the solute, the COSMO-RS method proved to be a reliable predictive tool for the prediction of partitioning for HMF, Fur, LA, and FA in a biphasic system, allowing for the identification of 11 solvents for evaluation of liquid–liquid extraction at 298 and 323 K. Furthermore, the use of HSP and RED generally indicates the favorability of solute–solvent dissolution over water solute. The combined approach of the COSMO-RS method, assessment of the EHS profile of solvents, and subsequent experimental validation successfully identified a selection of green solvents suitable for furan extraction from aqueous media with excellent partition capabilities. The attempts at reuse of isophorone, cyclohexanone, and MIBK proved positive, with stable partitioning across four total runs, indicating good stability of the solvent after 4 cycles with decreases of the extractive capability of less than 18% for all solutes, highlighting a decline of less than 7% in performance for MIBK in the extraction of Fur.

With an eye toward the future, thorough LLE studies with the identified systems will be conducted to acknowledge the effect of feed composition on the aqueous and organic phases, which is of utmost importance for process modeling of the combined reaction with *in situ* extraction for the biphasic production of HMF and Fur.

## Supporting Information Available

Solvents for COSMO-RS screening, EHS parameter evaluation, mutual solvent solubility data for partition experiments, result calibrations, thermodynamic contributions, Hansen solubility parameters, and VLE estimation with the NRTL model (PDF)

## Dedication

This work is dedicated to the loving memory of Carmelo Esteban. Thank you for everything.
