# Supplementary material for: Extraction of 5-Hydroxymethylfurfural and Furfural in Aqueous Biphasic Systems: A COSMO-RS Guided Approach to Greener Solvent Selection
Source: ACS Sustain Chem Eng. 2024 Feb 20;12(9):3766–79. doi: 10.1021/acssuschemeng.3c07894 (PMC10915861; doi:10.1021/acssuschemeng.3c07894)
Supplement: Supplementary file 1 — sc3c07894_si_001.pdf [file sc3c07894_si_001.pdf]

Extraction of 5-hydroxymethylfurfuralfural and furfural in aqueous biphasic systems: A COSMO-RS guided approach to greener solvent selection

Supporting Information

Dominik Soukup-Carne [a], Pablo López-Porfiri [a], *Felipe Sanchez Bragagnolo* [b], *Cristiano Soleo Funari* [c], Xiaolei Fan [a], María González-Miquel [d] *Jesús Esteban* [a*]

[a] Department of Chemical Engineering, The University of Manchester, Oxford Road, Manchester M13 9PL, United Kingdom

[b] Multidisciplinary Laboratory of Food and Health (LabMAS), School of Applied Sciences (FCA), University of Campinas (UNICAMP), Rua Pedro Zaccaria 1300, 13484-350 Limeira, SP, Brazil

[c] School of Agricultural Sciences, São Paulo State University, Av. Universitária 3780, Botucatu 18610-034, São Paulo, Brazil

[d] Department of Industrial Chemical Engineering and Environment, ETSI Industrial, Polytechnic University of Madrid, St. José Gutiérrez Abascal 2, Madrid 28006, Spain

*Corresponding author: E-mail address: jesus.estebanserrano@manchester.ac.uk (Jesús Esteban)

This Supporting Information contains:

Number of pages: 21

Number of tables: 5

Number of figures: 9

Contents

Existing studies using the COSMO-RS method for solvent selection for HMF and Fur. S3
COSMO-RS Miscibility gap screening S4
CHEM21 EHS classification S8
Solvent miscibility data S9
HPLC Calibration S10
Parity plots for experimental and COSMO-RS partition coefficients S11
Partition experiment in an aqueous biphasic system with triethylamine S13
HMF and Fur partitioning in literature S14
COSMO-RS driven thermodynamic contribution analysis at 323 K S15
In silico Hansen solubility parameter estimation S16
VLE estimation with NRTL S20
References S21

Existing studies using the COSMO-RS method for solvent selection for HMF and Fur.

Table S1: Summary of existing studies based on COSMO-RS guided solvent selection for the extraction of HMF and/or Fur.

Solute	Scale of study	Most effective solvent	Ref.
HMF	6000 initial solvent manually sorted to 110	*o*-propylphenol and *o*-isopropylphenol	[1]
HMF	2500 solvents with experimental validation of 50	3-chlorophenol	[2]
HMF/Fur	177 with the top 2 candidates experimentally validated	Ethyl acetate for HMF, Methyl propionate for Fur	[3]
HMF/Fur	2200 with 28 experimentally validated	3-chlorophenol	[4]
HMF/Fur	105 hydrophobic deep eutectic solvents (computational study only)	tetrabutylammonium bromide	[5]
Fur	10 hydrophobic deep eutectic solvents, experimental validation of 4	Tetrapentylammonium bromide	[6]
Fur	30 biobased solvents, 16 experimentally validated	Thymol	[7]
Fur	30 terpene-derived bio-carbonates (computational only study)	Thymol	[8]
Fur	108 hydrophobic deep eutectic constituents, (computational study only)	3–5-di-tertbutylcatechol	[9]

COSMO-RS Miscibility gap screening

Table S2: COSMO-RS method determination of binary LLE, water-solvent, at 298 K and 323 K.

Solvent Class	Count	Solvent	298 K	323 K
**Alcohols**	1	1,3-dimethoxy-2-propanol	No	Yes
	2	1,3-propanediol	No	No
	3	1-butanol	Yes	Yes
	4	1-decanol	Yes	Yes
	5	1-heptanol	Yes	Yes
	6	1-hexanol	Yes	Yes
	7	1-octanol	Yes	Yes
	8	1-pentanol	Yes	Yes
	9	2-butanol	Yes	Yes
	10	2-ethyl-1-hexanol	Yes	Yes
	11	2-methyl-2-butanol	Yes	Yes
	12	2-pentanol	Yes	Yes
	13	2-propanol	Yes	Yes
	14	3-methoxy-1,2-propanediol	No	No
	15	3-methoxy-3-methylbutanol	Yes	Yes
	16	benzyl alcohol	Yes	Yes
	17	dihydro terpineol_a	Yes	Yes
	18	dihydro terpineol_b	Yes	Yes
	19	dihydromyrcenol_b	Yes	Yes
	20	ethanol	No	No
	21	ethylene glycol	No	No
	22	furfuryl alcohol	Yes	Yes
	23	geraniol	Yes	Yes
	24	glycerol	No	No
	25	isobutanol	Yes	Yes
	26	isopentanol	Yes	Yes
	27	methanol	No	No
	28	nopol	Yes	Yes
	29	propanol	Yes	Yes
	30	propylene glycol	No	No
	31	2,2-dimethyl-4-hydroxymethyl-1,3-dioxolane	Yes	Yes
	32	tert-butanol	Yes	Yes
	33	tetrahydrofurfuryl alcohol	No	No
	34	α-terpineol	Yes	Yes
**Esters**	35	1,2-ethanediol diacetate	Yes	Yes
	36	4-methyl-2-pentylacetate	Yes	Yes
	37	acetic anhydride	Yes	Yes
	38	adipic acid diethyl ester	Yes	Yes
	39	benzoic acid phenylmethylester	Yes	Yes
	40	butyl laurate	Yes	Yes
	41	dectadecanoic acid	Yes	Yes
	42	dibutyl sebacate	Yes	Yes
	43	diethyl phthalate	Yes	Yes

	44	dihydroterpinyl acetate	Yes	Yes
	45	diisobutyl glutarate	Yes	Yes
	46	diisobutyl succinate	Yes	Yes
	47	dimethyl adipate	Yes	Yes
	48	dimethyl glutarate	Yes	Yes
	49	dimethyl phthalate	Yes	Yes
	50	dimethyl succinate	Yes	Yes
	51	ethyl acetate	Yes	Yes
	52	ethyl lactate	Yes	Yes
	53	ethyl laurate	Yes	Yes
	54	ethyl linoleate	Yes	Yes
	55	ethyl oleate	Yes	Yes
	56	ethyl palmitate	Yes	Yes
	57	ethyl succinate	Yes	Yes
	58	geranyl acetate	Yes	Yes
	59	glycerol triacetate	Yes	Yes
	60	isoamyl acetate	Yes	Yes
	61	isobutyl acetate	Yes	Yes
	62	isopropyl acetate	Yes	Yes
	63	isopropyl myristate	Yes	Yes
	64	isopropyl palmitate	Yes	Yes
	65	methyl abietate	Yes	Yes
	66	methyl acetate	No	Yes
	67	methyl formate	No	No
	68	methyl laurate	Yes	Yes
	69	methyl linoleate	Yes	Yes
	70	methyl linolenate	Yes	Yes
	71	methyl myristate	Yes	Yes
	72	methyl oleate	Yes	Yes
	73	methyl palmitate	Yes	Yes
	74	methyl propionate	Yes	Yes
	75	methyl stearate	Yes	Yes
	76	n-butyl acetate	Yes	Yes
	77	n-pentyl acetate	Yes	Yes
	78	n-propyl acetate	Yes	Yes
	79	oleyl alcohol	Yes	Yes
	80	tributylphosphate	Yes	Yes
	81	triethyl citrate	Yes	Yes
	82	γ-Valerolactone	No	No
**Ketones**	83	2-ethylhexyl acetate	Yes	Yes
	84	5-nonanone	Yes	Yes
	85	acetone	No	No
	86	acetophenone	Yes	Yes
	87	butanone	No	No
	88	cyclohexanoneoxime	Yes	Yes
	89	cyclopentanone	No	No
	90	isophorone	Yes	Yes
	91	methyl isobutyl ketone	Yes	Yes
**Organic acids**	92	acetic acid	No	No

	93	citric acid	No	No
	94	cyclohexanone	No	Yes
	95	formic acid	No	No
	96	hydracrylic acid	No	No
	97	lactic acid	No	No
	98	maleic acid	No	No
	99	Oleic acid	Yes	Yes
	100	propionic acid	No	No
**Ethers**	101	1,1,3-trimethoxypropane	Yes	Yes
	102	1,2-dimethoxyethane	No	No
	103	1,4-cineol	Yes	Yes
	104	1,8-cineol	Yes	Yes
	105	2-ethoxy-2-methyl-propane	Yes	Yes
	106	2-methoxyethanol	No	No
	107	2-methyltetrahydrofuran	Yes	Yes
	108	anisole	Yes	Yes
	109	cyclopentyl methyl ether	Yes	Yes
	110	diethoxymethane	Yes	Yes
	111	diethyl ether	Yes	No
	112	diglyme	No	Yes
	113	diisopropyl ether	Yes	Yes
	114	dimethoxymethane	No	Yes
	115	dimethyl ether	No	No
	116	dioxane	No	No
	117	dioxolane	No	No
	118	dipropyleneglycol	No	Yes
	119	methanesulfonic acid	No	No
	120	methyl tert-amyl ether	Yes	Yes
	121	methyl-t-butylether	Yes	Yes
	122	tetrahydrofuran	Yes	Yes
**Organic carbonates**	123	diethylcarbonate	Yes	Yes
	124	dimethylcarbonate	Yes	Yes
	125	glycerolcarbonate	No	No
	126	propylenecarbonate	Yes	Yes
**Dipolar aprotic**	127	2-nitropropane	Yes	Yes
	128	acetonitrile	No	No
	129	dimethylformamide	No	No
	130	dimethylsulfoxide	No	No
	131	n,n-dimethylacetamide	No	No
	132	n,n-dimethyldecanamide	Yes	Yes
	133	nitromethane	Yes	Yes
	134	sulfolane	No	No
**Aromatics**	135	1,2-dimethylbenzene	Yes	Yes
	136	1,3-dimethylbenzene	Yes	Yes
	137	1,4-dimethylbenzene	Yes	Yes
	138	benzene	Yes	Yes
	139	hexamethylphosphoramide	Yes	Yes
	140	toluene	Yes	Yes
**Hydrocarbons**	141	1-methyl-4-isopropylbenzene	Yes	Yes

	142	2,2,4-trimethylpentane	Yes	Yes
	143	cyclohexane	Yes	Yes
	144	hexane	Yes	Yes
	145	limonene	Yes	Yes
	146	methylcyclohexane	Yes	Yes
	147	myrcene	Yes	Yes
	148	n-heptane	Yes	Yes
	149	pentane	Yes	Yes
	150	terpinolene	Yes	Yes
	151	α-pinene	Yes	Yes
	152	β-pinene	Yes	Yes
**Halogenated**	153	1,1,2-trichloroethane	Yes	Yes
	154	1,2-dichloroethane	Yes	Yes
	155	chlorobenzene	Yes	Yes
	156	chloroform	Yes	Yes
	157	dichloromethane	Yes	Yes
	158	tetrachloromethane	Yes	Yes
	159	trifluoroacetic acid	No	No
	160	trifluoromethyl benzene	Yes	Yes
	161	β-farnesene	Yes	Yes
**Bases**	162	1,2-dimethyl-3-nitrobenzene	Yes	Yes
	163	1,3-dimethyl-2-imidazolidinone	No	No
	164	1,3-dimethyl-2-nitrobenzene	Yes	Yes
	165	1,4-dimethyl-2-nitrobenzene	Yes	Yes
	166	2,4,6-collidine	Yes	Yes
	167	2-pyrrolidone	No	No
	168	N,N′-Dimethylpropyleneurea	No	No
	169	n-methyl-2-pyrrolidinone	No	No
	170	perfluorooctane	Yes	Yes
	171	pyridine	Yes	Yes
	172	tetramethylurea	No	No
	173	triethylamine	Yes	Yes
**Other**	174	carbon disulfide	Yes	Yes
	175	decamethylcyclopentasiloxane	Yes	Yes
	176	isopropylphenol	Yes	Yes

CHEM21 EHS classification

Table S3: EHS based CHEM21 classification of solvents identified in the top 20 ranking of solvents for HMF and Fur extraction by the COSMO-RS method and those subsequently experimentally validated.

Solvent	Environmental score	Health score	Safety score	CHEM21 EHS classification
**1,1,2-trichloroethane**	1	6	3	Recommended
**1,2-ethanediol diacetate**	2	2	5	Recommended
**1,3-dimethoxy-2-propanol**	5	2	5	Problematic
**1-octanol**	2	2	7	Problematic
**2,4,6-collidine**	5	5	5	Problematic
**2-methyltetrahydrofuran (MTHF)**	6	5	3	Problematic
**3-methoxy-3-methylbutanol**	2	2	5	Recommended
**4-isopropylphenol**	6	7	7	Hazardous
**acetic anhydride**	4	7	5	Problematic
**acetophenone**	2	2	7	Problematic
**chloroform**	6	7	5	Problematic
**cyclopentyl methyl ether (CPME)**	7	2	5	Problematic
**cyclohexanone**	3	2	5	Recommended
**1,2-dichlorethane (DCE)**	5	10	3	Hazardous
**dichloromethane**	5	7	7	Hazardous
**diglyme**	4	9	5	Hazardous
**dimethoxymethane**	5	3	7	Problematic
**dimethyl adipate**	2	2	7	Problematic
**dimethyl glutarate**	2	1	7	Problematic
**dimethyl phthalate**	2	1	7	Problematic
**dimethyl succinate**	2	5	5	Problematic
**dimethylcarbonate (DMC)**	4	1	3	Recommended
**dipropyleneglycol**	2	1	7	Problematic
**ethyl lactate**	4	4	5	Problematic
**furfuryl alcohol**	3	6	5	Problematic
**glycerol triacetate**	2	1	7	Problematic
**hexamethylphosphoramide**	3	9	7	Hazardous
**isophorone**	3	6	7	Problematic
**methyl acetate**	5	3	5	Problematic
**Methyl isobutyl ketone (MIBK)**	4	2	3	Recommended
**n,n-dimethyldecanamide**	3	5	5	Problematic
**pyridine**	5	2	3	Recommended
**solketal**	9	2	7	Hazardous
**tetrahydrofuran**	6	7	5	Problematic
**triethyl citrate**	2	5	7	Problematic
**triethylamine**	6	7	3	Problematic

Solvent miscibility data

Table S4: solvent solubility data at 298 K and 323 K

Solvent	Solvent in water (g.ml^{-1})	Ref	Water in solvent (g.ml^{-1})	Ref.
		**298 K**		
1,2-ethanediol diacetate	0.1600	10	--	--
1-octanol	0.0005	11	0.0419	11
CPME	0.0110	12	0.0048	12
Cyclohexanone	0.0874	13	0.0592	13
DCE	0.0086	14	--	--
DMC	0.1390	15	0.0850	15
Isophorone	0.0120	16	--	--
MIBK	0.0184	17	0.0155	17
MTHF	0.1480	12	0.0311	12
Triethylamine	0.0812	18	--	--
		**323 K**		
1,2-ethanediol diacetate	0.1540	10	--	--
1-octanol	0.0011	11	0.0465	11
CPME	0.0076	12	0.0057	12
Cyclohexanone	0.0782	19	0.0725	19
DCE	--	--	--	--
DMC	0.1771	15	0.1320	15
Isophorone	--	--	--	--
MIBK	0.0139	20	0.0240	21
MTHF	0.0862	12	0.0327	12
Triethylamine	--	--	--	--

HPLC Calibration

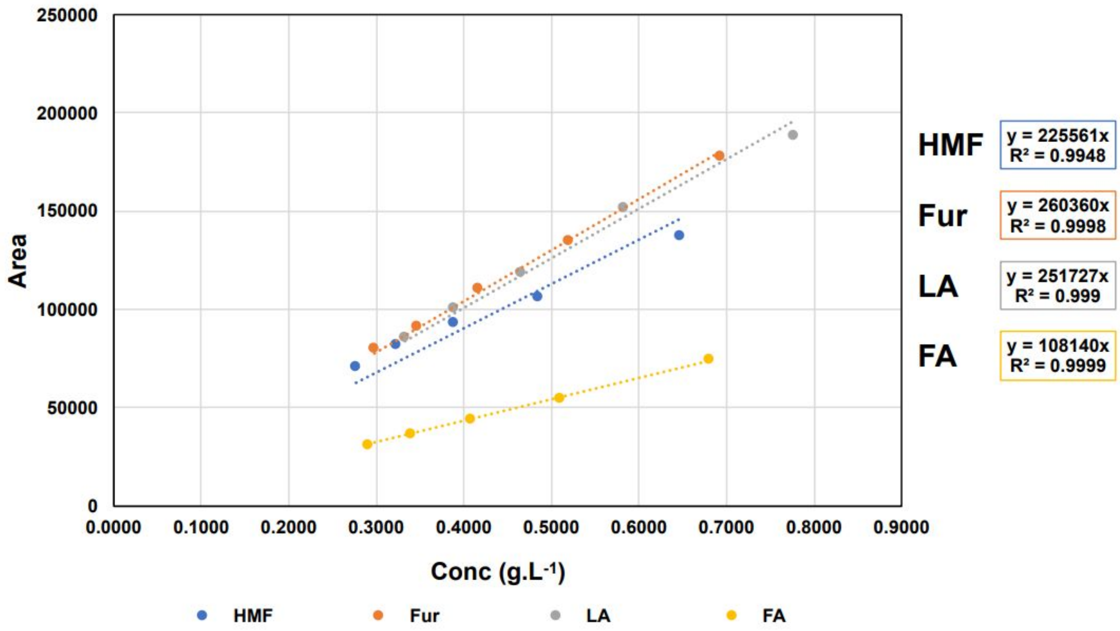

Figure S1: HPLC calibrations for HMF, Fur, LA, and FA. Where DAD used at 282 nm for HMF and 277 nm for Fur, RID used for LA and FA detection only.

Parity plots for experimental and COSMO-RS partition coefficients

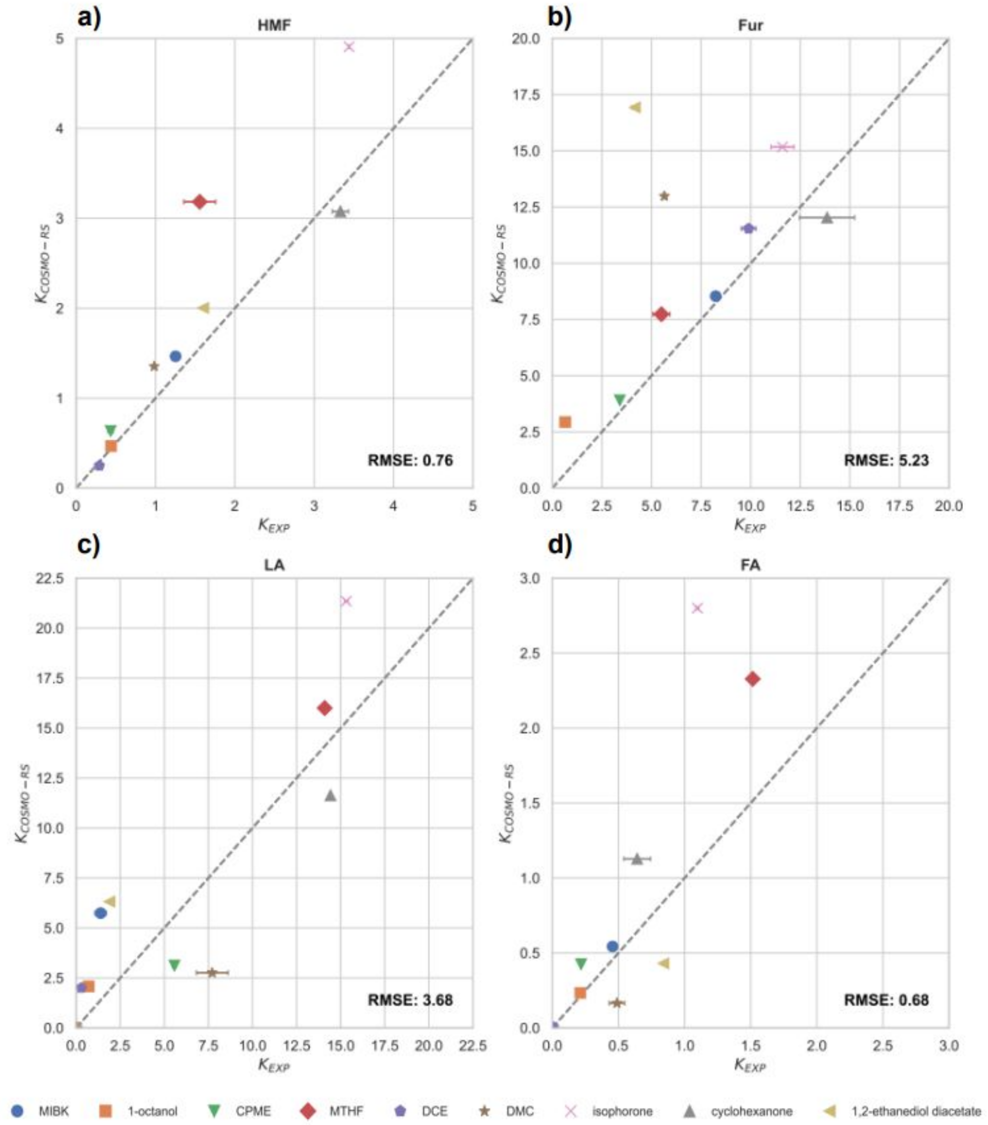

Figure S2: Comparison between experimentally observed and COSMO-RS predicted partition coefficients in the form of Parity plots at 298 K for a) HMF, b) Fur, c) LA, and d) FA.

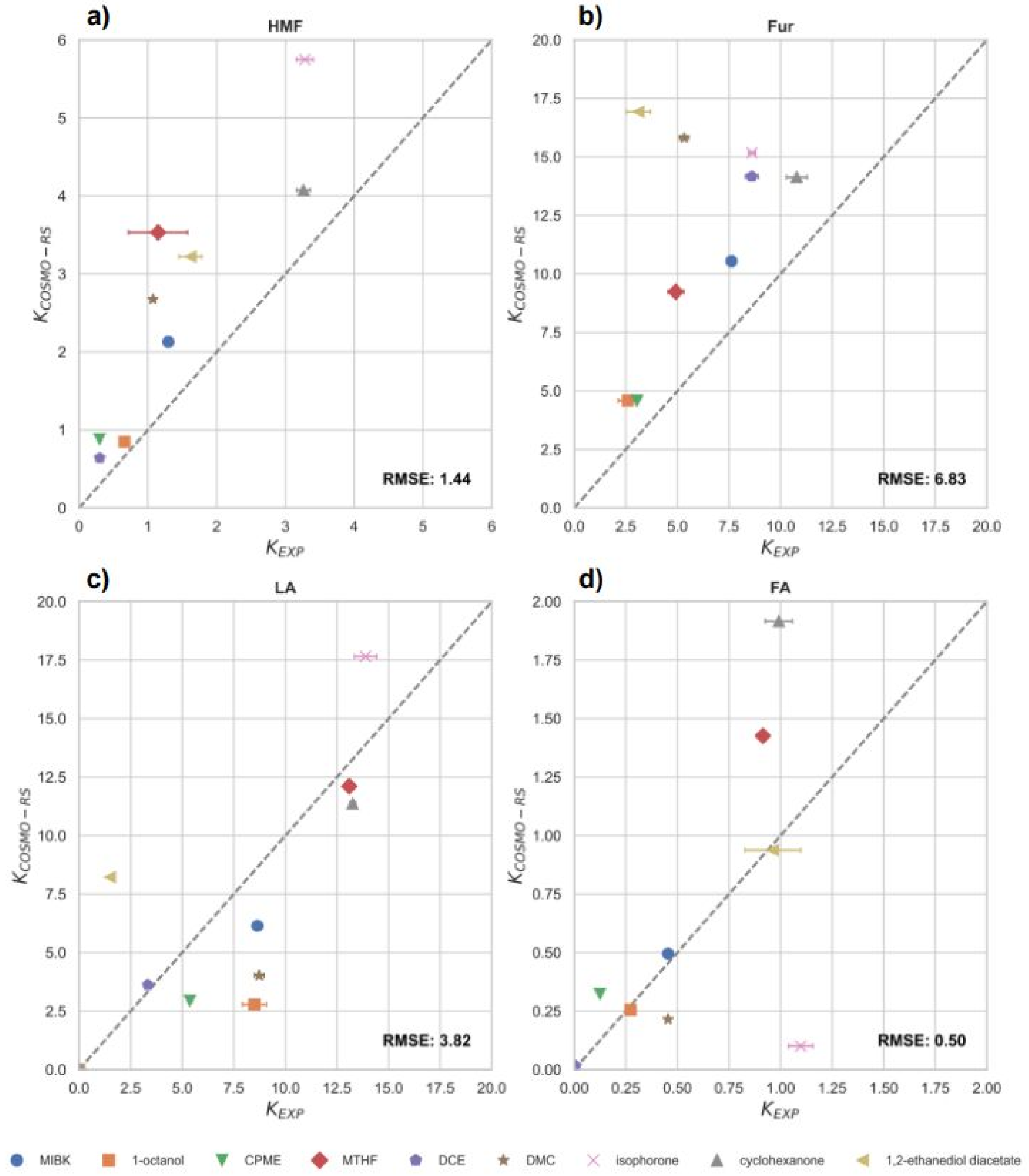

Figure S3: Comparison between experimentally observed and COSMO-RS predicted partition coefficients in the form of Parity plots at 323 K for a) HMF, b) Fur, c) LA, and d) FA.

Table S5: Experimentally determined and COSMO-RS predicted partition coefficients of HMF, Fur, LA, and FA with 4-isopropylphenol at 343 K.

Solute	K_{Exp}± SD	$K_{COSMO-RS}$
HMF	6.144 ± 0.018	13.359
Fur	20.669 ± 1.350	93.148
LA	0.280 ± 0.074	22.620
FA	0.106 ± 0.004	0.119

Partition experiment in an aqueous biphasic system with triethylamine

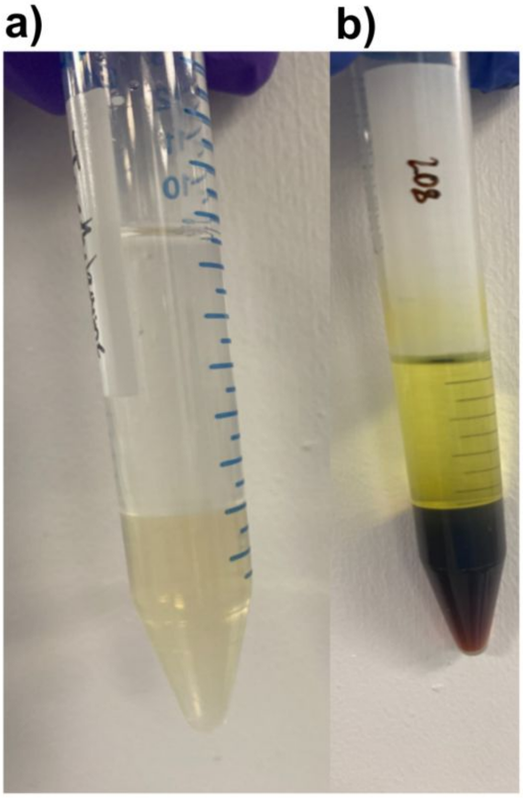

Figure S4: Partition experiment with triethylamine as the organic phase (upper), water phase (lower), with 1 wt.% HMF a) before partition experiment and b) after partition experiment at 323 K.

COSMO-RS driven thermodynamic contribution analysis at 323 K

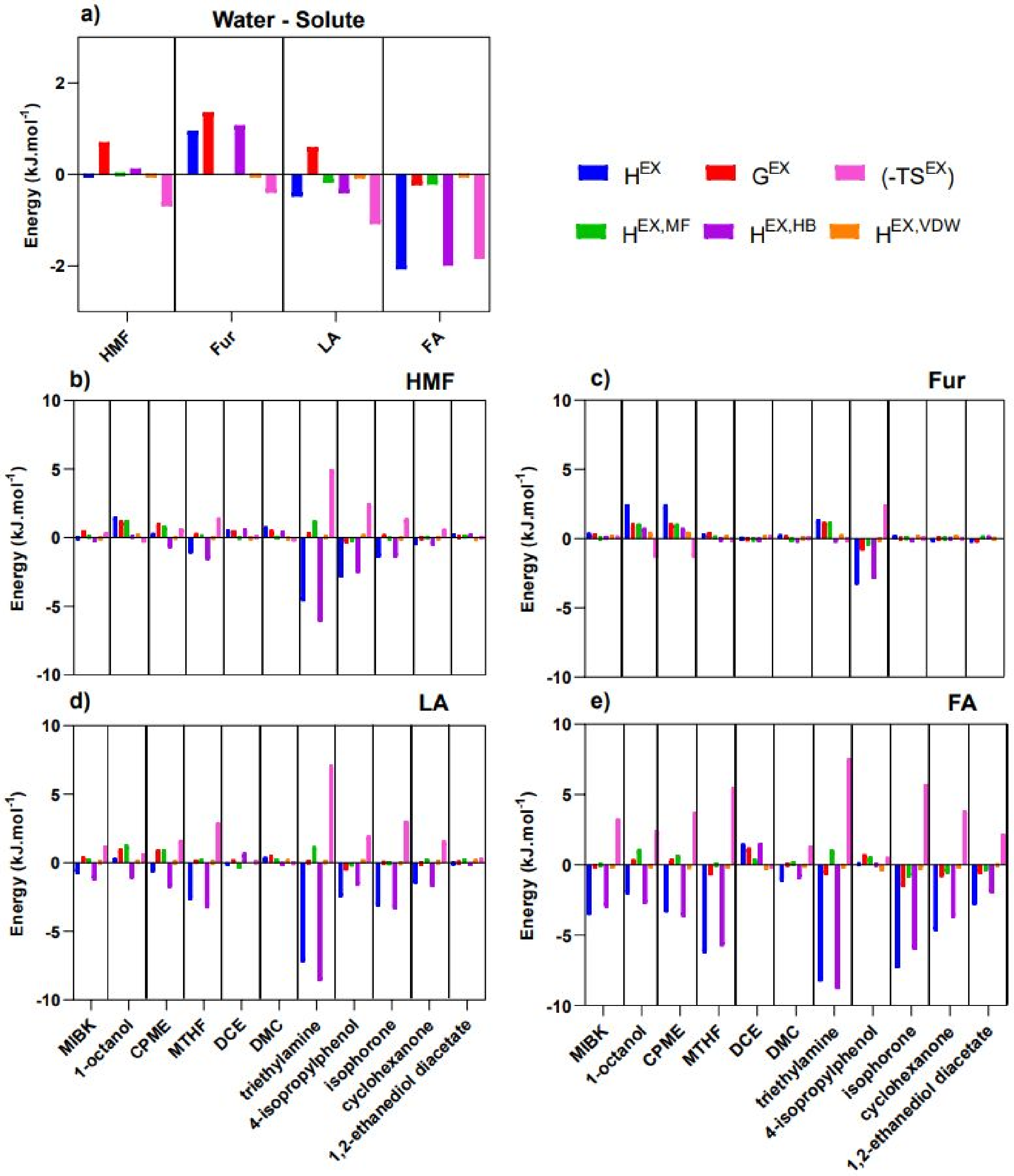

Figure S5: COSMO-RS calculations of the excess enthalpy (H^{EX}), with three constituent parts MF – misfit forces, HB- hydrogen bonding, VDW- Van Der Waals ,excess free energy (G^{EX}) and entropy ($-TS^{EX}$) at 323 K for binary mixtures of a) water-HMF, Fur, LA, and FA, b) HMF-solvent, c) Fur-solvent, d) LA-solvent and e) FA-solvent.

In silico Hansen solubility parameter estimation

Figure S6 presents the three calculated parameters, dispersion (δ_D), dipole moment (δ_P), and hydrogen bond interactions (δ_H) for HMF, Fur, LA, and FA at 298 K alongside the 11 studied solvents. The general premise of HSP is that similar values of HSP indicate favourable solute dissolution with said solvent, for example Fur with a δ_D of 18.6 and 18.0 for 4-isopropylphenol; in contrast, they have significant differences between their δ_P and δ_h. The major differences between the HSP of HMF and Fur were the magnitudes for δ_H which follows with HMF values at 14.1 versus the 5.1 for Fur. This is due to the lack of -OH moiety driving hydrogen bonding in HMF whilst the same general structure exists for both outside of this *viz* the aldehyde function and furan ring.

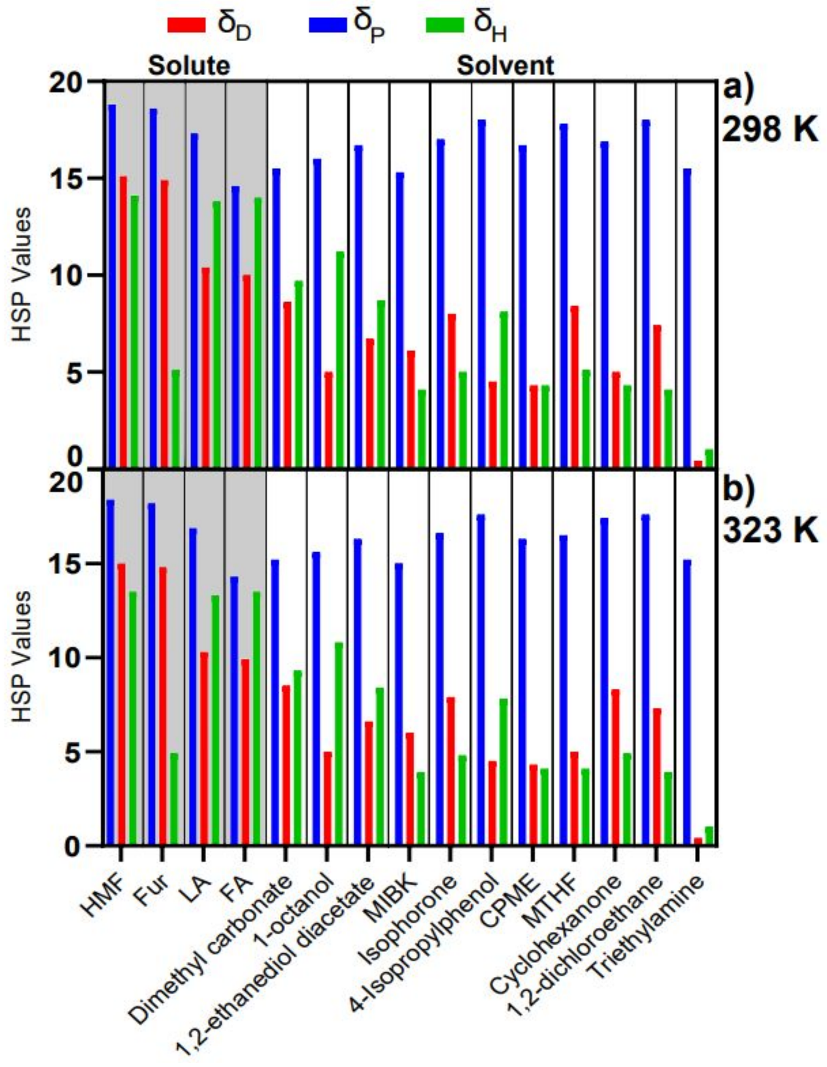

Figure S6: HSP (dispersion (δ_D), dipole moment (δ_P), and hydrogen bond interactions (δ_H)) for HMF, Fur, LA, and FA at a) 298 K and b) 323 K.

The similarities in HSP values between LA and FA are evident with similar values for δ_H at 13.8 and 14, δ_P of 10.4 and 10, respectively. The difference is that δ_D is attributed to the larger nature of LA hence more prevalence of London dispersion forces yielding a result of 17.3 compared to just 14.6 for FA. There is little significant difference in HSP at 298 K and 323 K due to the HSP being independent of temperature. These HSP can be used further to provide a definitive overall estimation of probability of solute solvation in a given system using RED, calculated with Equation 5. These RED values can be determined using the HSP, which calculates a value for R_a; however, the calculation of R_0 requires the use of a model based on assigning scores to a selection of known solvents. Typically, the assignment of scores or solvent rankings from 1-6, where 1 is the best solvent and 6 is the worst is done through eye and experimental testing to identify solubility [29, 30]. For this, ten representative recommended solvents, i.e., acetone, anisole, ethanol, ethyl acetate, IPA, methanol, methyl ethyl ketone, n-butyl alcohol, tert-amyl methyl ether, and water, had their respective activity coefficients at infinite dilution, ln(γ), generated by COSMO-RS and parameterized on a scale from 1-6, with the lowest ln(γ) represented by 1 and the highest by 6 [31]. These scores were then applied to the list of studied solvents to fit and generate associated R_0 values which could be used the calculated the RED, equation 7, and presented in Figure S7.

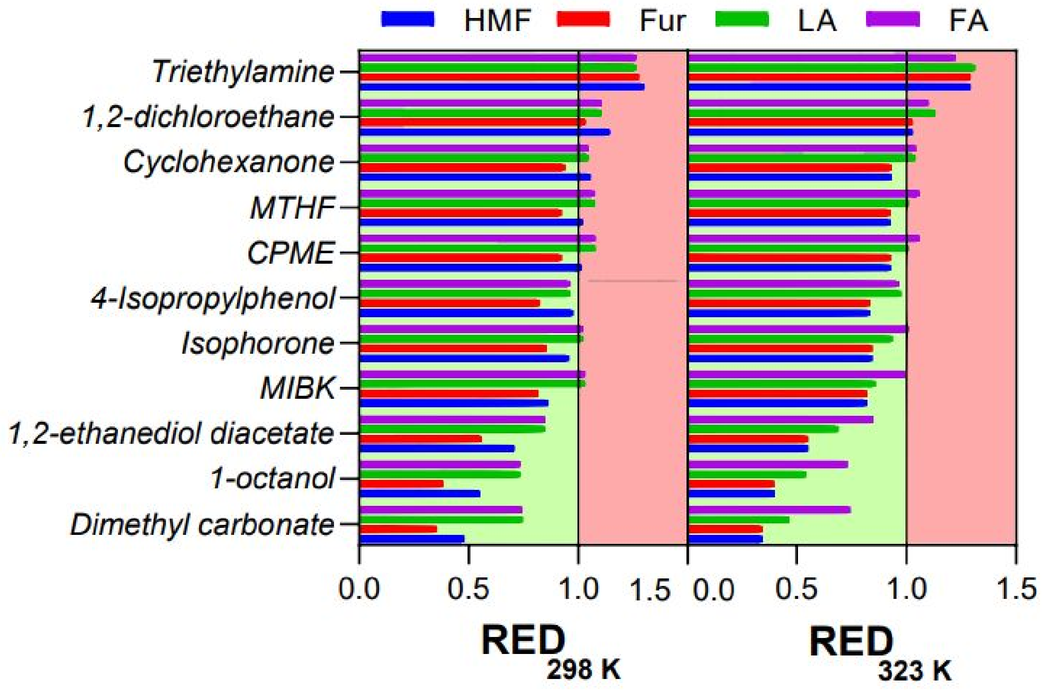

Figure S7: RED of HMF, Fur, LA, and FA at 298 K and 323 K, where the green shaded regions indicate favourable dissolution and red areas the opposite .

RED values indicate levels of solubility or likelihood that a solute will dissolve in a specific solvent. RED < 1 indicates high chance of dissolution, RED > 1 a lower chance, RED = 0 a perfect system where the solute instantaneously fully dissolves and RED = 1 where a moderate chance of dissolution is observed

[29]. The dissolution of HMF is estimated to occur is in approximately half of the solvents studied, although these predictions only display the likelihood or probability of dissolution not a discrete finite deduction. DMC is predicted to spontaneously dissolve HMF with a RED value <0.5, which aligns with Dibenedetto *et al*. and their use of DMC for extraction of HMF [32]. It is clear from Figure S7 that the majority of solvents, excluding triethylamine and 1,2-dichloroethane, were suitable for the dissolution of Fur. With both 1-octanol and DMC displaying excellent affinity for Fur with RED < 0.5 [33, 34]. The RED values of LA and FA are nearly identical due to the similar structure and method of calculation with respect to the scores ranking, thus can be discussed in tandem. Four solvents are predicted to have RED values less than unity at values of 0.736, 0.749, 0.852 and 0.964 for 1-octanol, DMC, 1,2-ethanediol diacetate and 4-isopropylphenol. Knowledge of these results help guide and validate experimental results. Comparing these RED results to those from COSMO-RS allows for identification of limitations and potential issues. The RED determined for HMF at 298 K with the partition coefficients of COSMO-RS, parallels and differences can immediately be identified. For example, the excellent prediction of HMF solubility in DMC with HSP is reflected by a relatively average K_{HMF} of 1.35 with COSMO-RS. Fur dissolution is predicted to be extremely high in 1-octanol, due to the large degree of similarity in the non-polarity of both molecules; however, the COSMO-RS partition predictions are fairly at 2.94. DMC is estimated by RED to be also an excellent extractant for Fur, which is supported by the COSMO-RS K_{Fur} of 12.98. Interestingly, triethylamine is predicted to have poor dissolution of all solutes yet is consistently predicted to possess the highest extraction capability by at least an order of magnitude in each scenario. Across the range of studied solvents, the dissolution of both LA and FA is considerably lower than those of HMF and Fur, owing to the increased polarity of said organic acids over the non-polarity of the furans. The RED value decreases with respect to temperature in general across all solutes and solvents indicating the influence and favourable of dissolution at higher temperatures. Figure S8 presents the RED calculation against the experimental solubility of solutes, HMF, Fur, LA and FA, in a range of studied solvents at (Figure S8a) 298 K and (Figure S8b) 323 K.

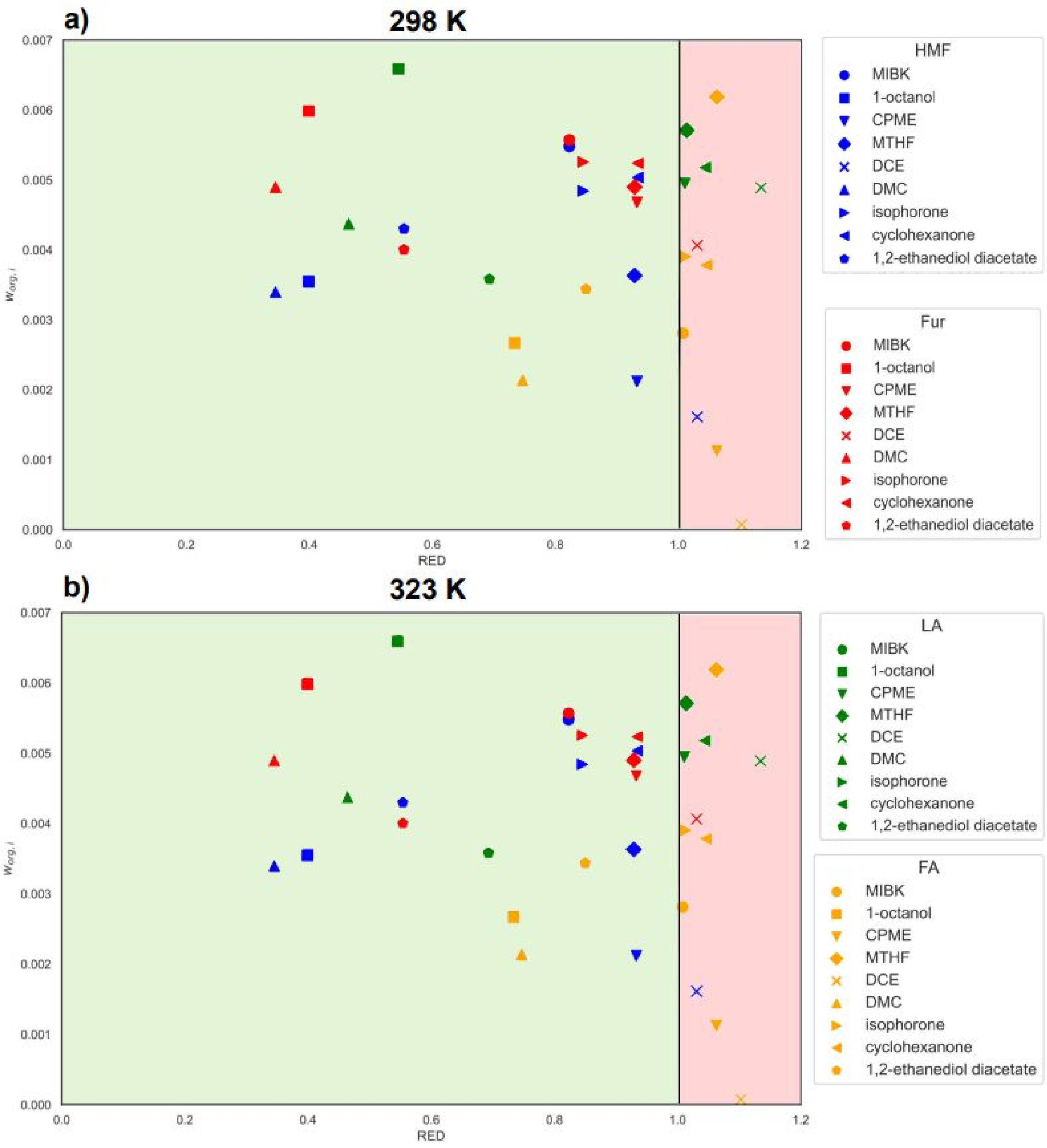

Figure S8: RED vs solubility of HMF, Fur, LA, and FA in organic phase for a) 298 K and b) 323 K, where green shaded region represents favourable probability of dissolution and red unfavourable dissolution.

VLE estimation with NRTL

Prior to solvent reuse, ASPEN Plus v12.0 was used to practically implement the NRTL model to estimate VLE of HMF and Fur with MIBK, cyclohexanone and isophorone. These estimations taken over 200 intervals of mass fractions at 101.3 kPa were used to predict formation of azeotropes that may hinder vacuum distillation for solvent reuse capabilities. The six generated VLE are presented in Figure S9 and show that no azeotropes are present in the studied systems.

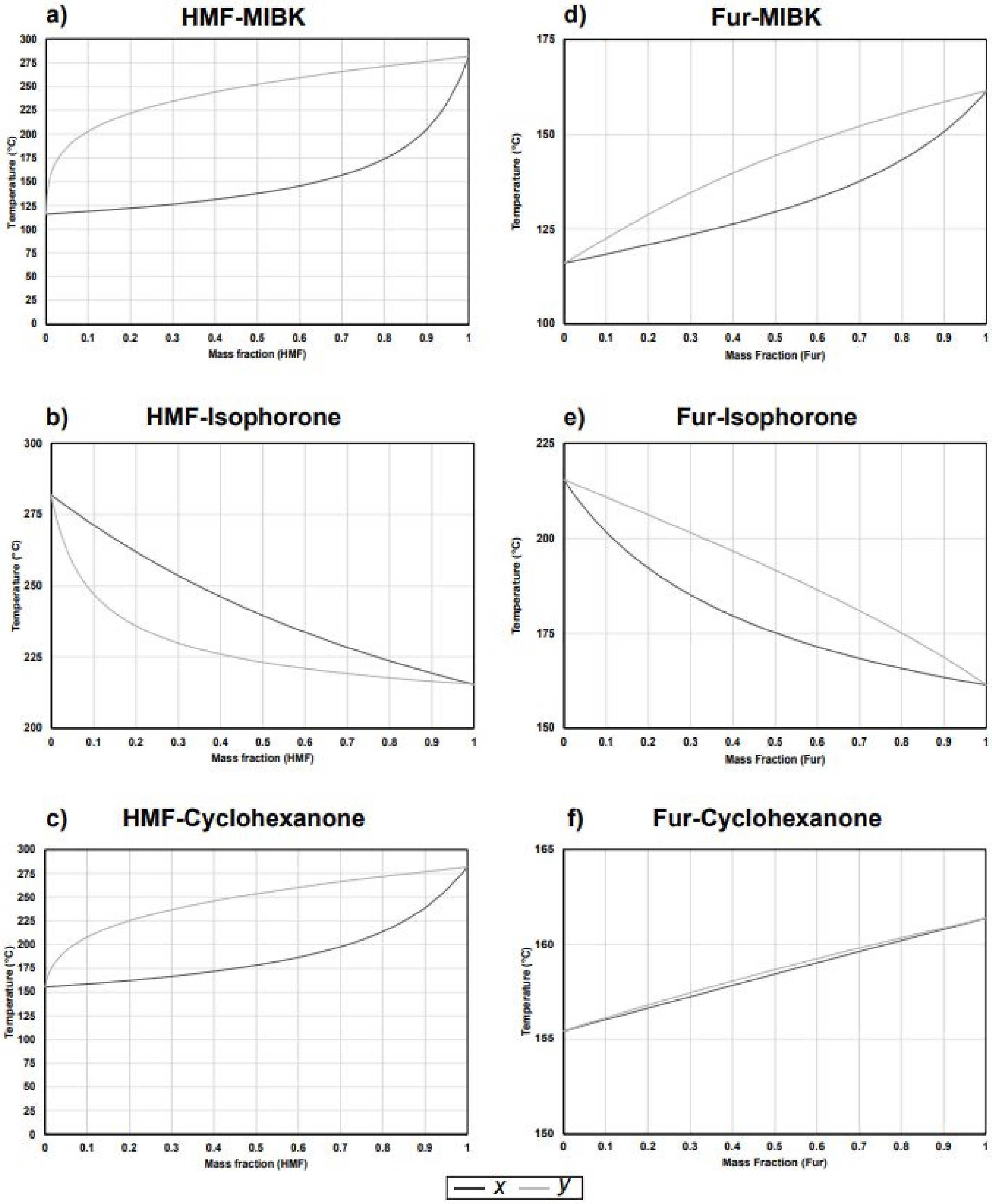

Figure S9: VLE estimation using the NRTL model applied in ASPEN Plus v12.0 at 101.3 kPa for HMF with a) MIBK, b) isophorone c) cyclohexanone and for FUR with d) MIBK, e) isophorone f) cyclohexanone.

References

(1) Blumenthal, L. C.; Jens, C. M.; Ulbrich, J.; Schwering, F.; Langrehr, V.; Turek, T.; Kunz, U.; Leonhard, K.; Palkovits, R., Systematic Identification of Solvents Optimal for the Extraction of 5-Hydroxymethylfurfural from Aqueous Reactive Solutions. *ACS Sustain. Chem. Eng.* **2016,** *4* (1), 228-235.

(2) Wang, Z.; Bhattacharyya, S.; Vlachos, D. G., Solvent selection for biphasic extraction of 5-hydroxymethylfurfural via multiscale modeling and experiments. *Green Chem.* **2020,** *22* (24), 8699-8712.
(3) Esteban, J.; Vorholt, A. J.; Leitner, W., An overview of the biphasic dehydration of sugars to 5-hydroxymethylfurfural and furfural: a rational selection of solvents using COSMO-RS and selection guides. *Green Chem.* **2020,** *22* (7), 2097-2128.
(4) Wang, Z. X.; Bhattacharyya, S.; Vlachos, D. G., Extraction of Furfural and Furfural/5-Hydroxymethylfurfural from Mixed Lignocellulosic Biomass-Derived Feedstocks. *ACS Sustain. Chem. Eng.* **2021,** *9* (22), 7489-7498.
(5) Quaid, T.; Reza, T., COSMO-RS predictive screening of type 5 hydrophobic deep eutectic solvents for selective platform chemicals absorption. *J. Mol. Liq.* **2023,** *382*.
(6) McGaughy, K.; Reza, M. T., Liquid-Liquid Extraction of Furfural from Water by Hydrophobic Deep Eutectic Solvents: Improvement of Density Function Theory Modeling with Experimental Validations. *ACS Omega* **2020,** *5* (35), 22305-22313.
(7) Canada-Barcala, A.; Rodriguez-Llorente, D.; Lopez, L.; Navarro, P.; Hernandez, E.; Agueda, V. I.; Alvarez-Torrellas, S.; Parajo, J. C.; Rivas, S.; Larriba, M., Sustainable Production of Furfural in Biphasic Reactors Using Terpenoids and Hydrophobic Eutectic Solvents. *ACS Sustain. Chem. Eng.* **2021,** *9* (30), 10266-10275.
(8) Belinchón, A.; Hernández, E.; Vázquez, J.; Santiago, R.; Moya, C.; Larriba, M.; Navarro, P.; Palomar, J., Biocarbonates Derived from CO2 and Terpenes: Molecular Design for Aqueous Mixture Treatment Driven by COSMO-RS. *ACS Sustain. Chem. Eng.* **2022,** *10* (29), 9635-9643.
(9) Darwish, A. S.; Lemaoui, T.; AlYammahi, J.; Taher, H.; Benguerba, Y.; Banat, F.; AlNashef, I. M., Molecular insights into potential hydrophobic deep eutectic solvents for furfural extraction guided by COSMO-RS and machine learning. *J. Mol. Liq.* **2023,** *379*.
(10) Browning, E., Toxicity and metabolism of industrial solvents. *Toxicity and Metabolism of Industrial Solvents.* **1965**.
(11) Ebrahimkhani, M. J.; Azadian, M.; Ghanadzadeh Gilani, H., Experimental Data and Thermodynamic Modeling of the Liquid–Liquid Equilibrium Ternary System (Water + Acetic Acid + 1-Octanol) at Several Temperatures. *Journal of Chemical & Engineering Data* **2022,** *67* (2), 404-415.
(12) Männistö, M.; Pokki, J.-P.; Fournis, L.; Alopaeus, V., Ternary and binary LLE measurements for solvent (2-methyltetrahydrofuran and cyclopentyl methyl ether)+furfural+water between 298 and 343K. *J. Chem. Thermodyn.* **2017,** *110*, 127-136.
(13) Çehreli, S.; Tatlí, B.; Bagˇman, P., (Liquid+liquid) equilibria of (water+propionic acid+cyclohexanone) at several temperatures. *J. Chem. Thermodyn* **2005,** *37* (12), 1288-1293.
(14) McGovern, E. W., Chlorohydrocarbon Solvents. *Industrial & Engineering Chemistry* **1943,** *35* (12), 1230-1239.
(15) de la Torre, J.; Cháfer, A.; Berna, A.; Muñoz, R., Liquid–liquid equlibria of the system dimethyl carbonate+methanol+water at different temperatures. *Fluid Phase Equilib.* **2006,** *247* (1), 40-46.
(16) Colombo, A.; Battilana, P.; Ragaini, V.; Bianchi, C. L.; Carvoli, G., Liquid−Liquid Equilibria of the Ternary Systems Water + Acetic Acid + Ethyl Acetate and Water + Acetic Acid + Isophorone (3,5,5-Trimethyl-2-cyclohexen-1-one). *Journal of Chemical & Engineering Data* **1999,** *44* (1), 35-39.
(17) Conway, J. B.; Philip, J. B., Ternary System: Furfural-Methyl Isobutyl Ketone-Water at 25 °C. *Industrial & Engineering Chemistry* **1953,** *45* (5), 1083-1085.
(18) Mendonça, Â. F. S. S.; Pereira, S. N. R.; Lampreia, I. M. S., Solubility of Triethylamine in Calcium Chloride Aqueous Solutions from 20 to 35°C. *J. Solution Chem.* **2003,** *32* (12), 1033-1044.
(19) Góral, M.; Wiśniewska-Gocłowska, B., IUPAC-NIST Solubility Data Series. 86. Ethers and Ketones with Water. Part 5. C6 Ketones with Water. *J. Phys. Chem. Ref. Data* **2008,** *37* (3), 1575-1609.
(20) Vakili-Nezhaad, G. R.; Mohsen-Nia, M.; Taghikhani, V.; Behpoor, M.; Aghahosseini, M., Salting-out effect of NaCl and KCl on the ternary LLE data for the systems of (water+propionic

acid+isopropyl methyl ketone) and of (water+propionic acid+isobutyl methyl ketone). *J. Chem. Thermodyn* **2004,** *36* (4), 341-348.
(21) Stephenson, R. M., Mutual solubilities: water-ketones, water-ethers, and water-gasoline-alcohols. *Journal of Chemical & Engineering Data* **1992,** *37* (1), 80-95.
(22) Mohammad, S.; Held, C.; Altuntepe, E.; Köse, T.; Sadowski, G., Influence of Salts on the Partitioning of 5-Hydroxymethylfurfural in Water/MIBK. *J. Phys. Chem. B* **2016,** *120* (16), 3797-3808.
(23) Zhang, Y. Z.; Guo, X.; Xu, J.; Wu, Y. X.; Lu, M. Z., Liquid-Liquid Equilibrium for Ternary Systems, Water+5-Hydroxymethylfurfural + (1-Butanol, Isobutanol, Methyl Isobutyl Ketone), at 313.15, 323.15, and 333.15 K. *J. Chem. Eng. Data* **2018,** *63* (8), 2775-2782.
(24) Zilnik, L. F.; Crnomarkovic, M.; Novak, U.; Grilc, M.; Likozar, B., Modelling, optimal solvent screening and separation of 5-hydroxymethylfurfural or furfural from catalytic conversion reactor stream in downstream purification process. *Chem. Eng. Res. Des.* **2023,** *194*, 376-387.
(25) Roth, D. M.; Haas, M.; Echtermeyer, A.; Kaminski, S.; Viell, J.; Jupke, A., The Effect of Sulfate Electrolytes on the Liquid–Liquid Equilibrium of 2-MTHF/Water/5-HMF: Experimental Study and Thermodynamic Modeling. *Journal of Chemical & Engineering Data* **2023,** *68* (6), 1397-1410.
(26) Sayed, M.; Warlin, N.; Hulteberg, C.; Munslow, I.; Lundmark, S.; Pajalic, O.; Tuna, P.; Zhang, B. Z.; Pyo, S. H.; Hatti-Kaul, R., 5-Hydroxymethylfurfural from fructose: an efficient continuous process in a water-dimethyl carbonate biphasic system with high yield product recovery. *Green Chem.* **2020,** *22* (16), 5402-5413.
(27) Mannisto, M.; Pokki, J. P.; Creati, A.; Voisin, A.; Zaitseva, A.; Alopaeus, V., Ternary and Binary LLE Measurements for Solvent (4-Methyl-2-pentanone and 2-Methyl-2-butanol) + Furfural plus Water between 298 and 401 K. *J. Chem. Eng. Data* **2016,** *61* (2), 903-911.
(28) Ershova, O.; Pokki, J.-P.; Zaitseva, A.; Alopaeus, V.; Sixta, H., Vapor pressure, vapor-liquid equilibria, liquid-liquid equilibria and excess enthalpy of the system consisting of isophorone, furfural, acetic acid and water. *Chem. Eng. Sci.* **2018,** *176*, 19-34.
(29) Milescu, R. A.; Segatto, M. L.; Stahl, A.; McElroy, C. R.; Farmer, T. J.; Clark, J. H.; Zuin, V. G., Sustainable Single-Stage Solid–Liquid Extraction of Hesperidin and Rutin from Agro-Products Using Cyrene. *ACS Sustain. Chem. Eng.* **2020,** *8* (49), 18245-18257.
(30) del Pilar Sánchez-Camargo, A.; Pleite, N.; Herrero, M.; Cifuentes, A.; Ibáñez, E.; Gilbert-López, B., New approaches for the selective extraction of bioactive compounds employing bio-based solvents and pressurized green processes. *J. Supercrit. Fluids* **2017,** *128*, 112-120.
(31) Hansen, C. M. A. S., *Hansen Solubility Parameters in Practice*. Hansen-Solubility: 2008.
(32) Dibenedetto, A.; Aresta, M.; di Bitonto, L.; Pastore, C., Organic Carbonates: Efficient Extraction Solvents for the Synthesis of HMF in Aqueous Media with Cerium Phosphates as Catalysts. *ChemSusChem* **2016,** *9* (1), 118-125.
(33) Raut, S. U.; Bhagat, P. R., Efficient photocatalytic acetalization of furfural to biofuel components using carboxyl-functionalized porphyrin photocatalyst, under visible light irradiations. *Biomass Conversion and Biorefinery* **2021**.
(34) Brouwer, T.; Blahusiak, M.; Babic, K.; Schuur, B., Reactive extraction and recovery of levulinic acid, formic acid and furfural from aqueous solutions containing sulphuric acid. *Sep. Purif. Technol.* **2017,** *185*, 186-195.
